# *Dynamozones* are the most obvious sign of the evolution of conformational dynamics in HIV-1 protease

**DOI:** 10.1038/s41598-023-40818-x

**Published:** 2023-08-30

**Authors:** Mohammad Rahimi, Majid Taghdir, Farzane Abasi Joozdani

**Affiliations:** Department of Biophysics, Faculty of Biological Science, Tarbiat Modares University, Tehran, 14115_111 Iran

**Keywords:** Biochemistry, Biophysics

## Abstract

Proteins are not static but are flexible molecules that can adopt many different conformations. The HIV-1 protease is an important target for the development of therapies to treat AIDS, due to its critical role in the viral life cycle. We investigated several dynamics studies on the HIV-1 protease families to illustrate the significance of examining the dynamic behaviors and molecular motions for an entire understanding of their dynamics-structure–function relationships. Using computer simulations and principal component analysis approaches, the dynamics data obtained revealed that: (i) The flap regions are the most obvious sign of the evolution of conformational dynamics in HIV-1 protease; (ii) There are dynamic structural regions in some proteins that contribute to the biological function and allostery of proteins via appropriate flexibility. These regions are a clear sign of the evolution of conformational dynamics of proteins, which we call dynamozones. The flap regions are one of the most important dynamozones members that are critical for HIV-1 protease function. Due to the existence of other members of dynamozones in different proteins, we propose to consider dynamozones as a footprint of the evolution of the conformational dynamics of proteins.

## Introduction

Proteins are biomolecules that are regarded as the machinery of life. They are intrinsically dynamic, and their conformational variability is essential to their biological functions^1,2^. The function of a protein besides the structure also relies on its dynamics. Protein flexibility is necessary for biological function, ligand binding, and protein–protein or protein-nucleic acid interactions. A quantitative description of protein dynamics is essential for understanding living systems at a molecular level and probably also for the mechanisms leading to protein malfunction^3,4^.

Protein flexibility refers to the protein structure's internal dynamics, which is beneficial in the structural and functional aspects of proteins^5^. Plasticity and Conformational mobility represent key intrinsic features of proteins through evolution. Internal mobility eases the evolution of proteins to adopt conformational flexibility and thus provides the opportunity to develop novel functions. In addition, conformational flexibility allows proteins to better cope with harmful mutations which can lead to loss of function or altered function that result in disease^5,6^. Despite substantial proof that suggests that protein dynamics are under evolutionary selection, little is known about the molecular basis of the evolution of protein dynamics or how they affect function. An interesting case in the context of the dynamic-function relationship is that structural dynamics play a significant role in protein promiscuity, which almost means the ability of proteins to carry out several more or less related molecular works.

Indeed, every protein has the potential to accept many various conformations in the native state, so, many proteins are capable to perform several functions^7,8^. The structural diversity linked to protein flexibility constitutes a basis of protein evolvability^6^. In some cases, the protein exhibits one well-defined primary function together with several low-level “promiscuous activities.” Moonlighting proteins are other cases that can effectively carry out several relevant functions or even different duties linked to various molecular surfaces or active sites. Thus, it appears rational to attend that a particular trait of dynamics in functionally related protein regions (e.g., conformational variety, active-site “flexibility”, or “deformability”, results in the ability to stabilize different substrates, transition states of leaving groups) may be linked to many cases of protein promiscuity^9,10^.

Protein conformational dynamics play an important role in evolution, normal physiology, and pathophysiology. The evolution of proteins involves mutations that may lead to proteins adopting new functions and, in rare cases, new folds. Indeed, mutations of proteins can alter their conformations, dynamics, and stability, and thereby play critical roles in evolution and diseases. At a molecular level, protein evolution is dominated by neutral or nearly neutral mutations that have little effect on function^11^. Nonetheless, our comprehension of how proteins and species evolve is still elementary. There is a lack of detailed understanding of how proteins have evolved^5,6^.

One of the important questions about the evolution of proteins that should be further investigated is: How do conformational dynamics evolve as proteins evolve? The results of some studies show that the dynamics and the evolution of proteins share similarities^12,13^. Tang et al.^14^ found that there is a correspondence between the dynamics and evolution of protein structures. Their results show that the evolutionary mechanism of the proteins obtains both dynamical flexibility and evolutionary structural variation. Studying the evolution of protein conformational dynamics would not be possible without the use of computational and biophysical methodologies, that allow structural dynamics to be dissected in different protein variants. Much work has been done on the evolution of protein structure, but the role of protein dynamics in evolution has received attention in recent years.

Molecular dynamics (MD) simulations are a powerful tool to investigate the dynamic behavior of proteins in an aqueous solution and deepen our understanding of the relationship between protein structure and function^3^. The global molecular motions of the proteins can be obtained by applying the combination of MD simulation and essential dynamics (ED) analysis technique^15^.

HIV-1 protease (human immunodeficiency virus type 1 protease), an aspartyl protease, is responsible for the generation of structural proteins and viral enzymes critical to HIV viral maturation and infectivity. Thus, HIV-1 protease is a major drug target in the battle against HIV-1 infection, where the inactivation of the HIV-1 protease causes the production of immature, noninfectious viral particles^16,17^. There are about 750 experimentally determined available structures of this enzyme and this wide structural knowledge allows a study of a large number of conformations of protease complexes. The HIV-1 protease is one of the best-characterized cases of protein molecular evolution^18^. The HIV-1 protease is a homodimer with two identical monomers (chain A and chain B) each consisting of 99 amino acids. Each monomer has one α-helix (usually residues from 86 to 90) and nine β-sheets in the secondary structure. The residues of HIV-1 protease are numbered 1–99 and 100–198 (or 1′–99′) for chains A and B, respectively.

**Figure 1:**
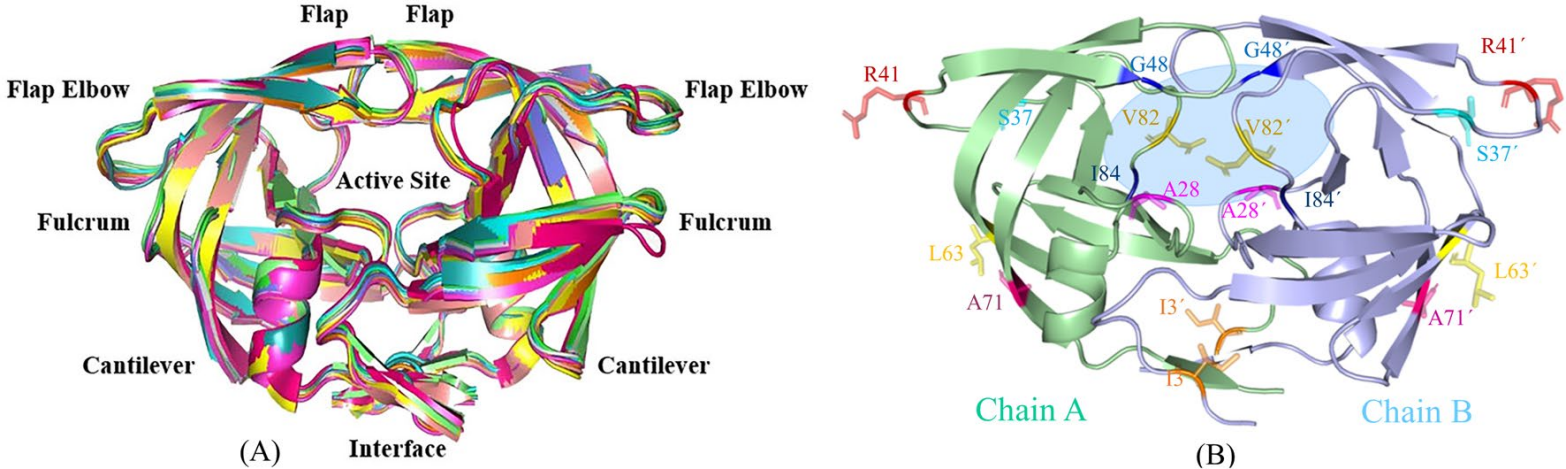
(**A**) Superposition of the crystal structures of the eleven selected HIV-1 proteases. Important regions of the HIV-1 proteases are labeled. (**B**) 3D structure of HIV-1 Protease (PDB code: 1HVI) showing the positions of mutated residues and also ligand position (light blue ellipse). Rendered using PyMOL.

HIV-1 protease is a consisting of six structural segments (Fig. 1A): interface (residues 1–5/100–104, 95–99/194–198), fulcrum (residues 11–22/110–121), active site (residues 23–30/122–129), flap (residues 43–58/142–157), flap elbow (residues 35–42/134–141), and cantilever (residues 59–75/158–174).

The active site of the enzyme is formed at the dimer interface with each monomer a conserved catalytic triad (Asp25-Thr26-Gly27). It’s gated by two extended β-hairpin loops known as flaps. Two Asp25 residues (one from each monomer or chain) act as the catalytic residues and the conserved active site residues forms a symmetrical and highly hydrogen-bonded arrangement^19,20^.

Investigating protein flexibility may be important for the study of processes associated with conformational changes and state transitions^21^. Structural and dynamical studies of the HIV-1 protease normally focused on its more flexible region, the flaps, since they control the entrance/stabilization of ligands in the active site^22,23^. There is a large variety in the flap conformations in the unbound state, fluctuating between the closed, semi-open, and wide-open conformations^24,25^. In the closed/semi-open state, the catalytic site is shielded with two flaps and thus limits the entry of most of the ligands. The semi-open conformation is the dominant state in the ligand-free HIV-1 protease^24–26^. The flexibility of the flap is needed to facilitate the substrate access to and product release from the active site of the enzyme by an open and close mechanism^27^. The binding of protease substrate to the active site can be controlled by limiting the movement of the flap, thereby inhibiting HIV-1 protease activity^24,28^.

In order to indicate the relationship between the dynamics of proteins and their structure properties, we examine several dynamics studies. This study includes the investigations of the molecular motions and dynamic behaviors of the HIV-1 protease family in relation to their structure using computer simulation techniques. We adopted here the unbound form of the proteases of HIV-1 to investigate their dynamics-structure–function relationships. We focus mainly on the following facets: (i) dynamic behavior and collective motions of the HIV-1 protease family; (ii) the effect of point mutations on the molecular motions and stability of the HIV-1 protease family; (iii) correlation of some dynamic structural regions with the evolution of conformational dynamics in the HIV-1 proteases family.

The cross-correlation analysis and principal component analysis (PCA) were also performed to probe the difference in internal dynamics and conformational changes of the selected proteins induced by mutation. It is evident from the correlation map that almost in all proteases the flaps and flap elbow motions are highly correlated.

## Results and discussion

### Sequence analysis

**Figure 2:**
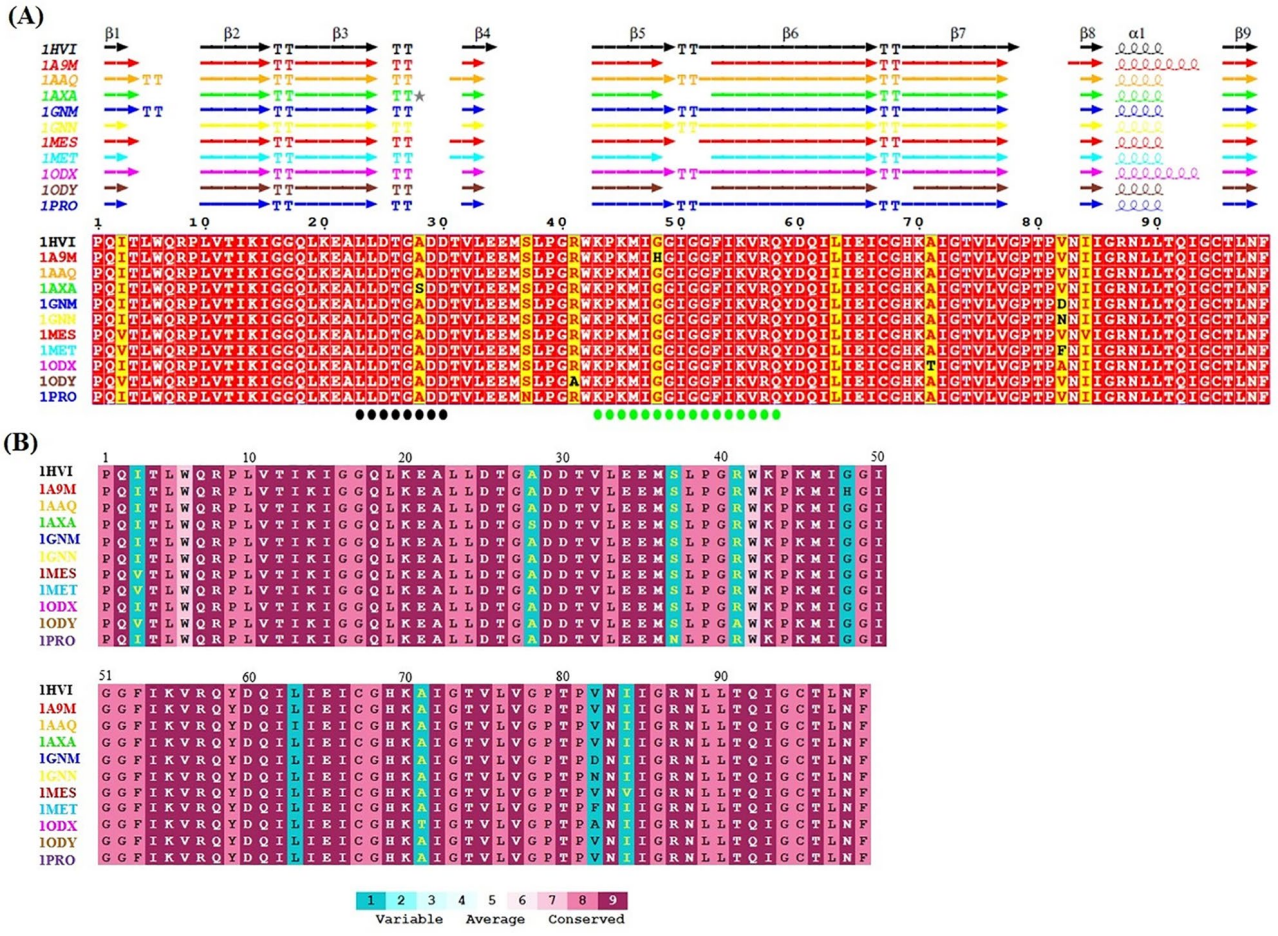
(**A**) Sequence alignment produced by T-COFFEE and the figure was prepared with ESPript 3.0. Identical residues are written in white bold characters and boxed in red whereas similar residues are in red bold characters and boxed in yellow. Secondary structure elements related to each protein are indicated at the top of sequence blocks (α, α-helix; β, β-strand; TT, turn). Residues forming the active site (23–30) and the flap region (43–58) are labelled with a black circle and a green circle under the sequence alignment, respectively. (**B**) The Evolutionary Conservation prediction analysis of amino acid residues of all the selected proteins by Consurf server. The amino acids are colored according to their conservation grades and conservation levels. A grade of 1 indicates rapidly evolving sites, which are color-coded in turquoise; 5 shows sites that are evolving at an average rate, which are colored white; and 9 shows evolutionarily conserved sites, which are color-coded in maroon.

Proteins with sequence identity > 30% typically belong to the same family and have similar conformation and function. Such clear homologues are probably to have separated from a common ancestor and their sequences may show conserved differences between species of organisms. The simultaneous comparison of sequence and structure information is of significance to detect biological specificities in a group of proteins^29,30^. Multiple sequence alignment was performed using T-COFFEE and rendered by ESPript 3.0 using default parameters for residue similarity calculations, where boxed residues represent identical (red box, white character) and similar (Yellow box, red character) amino acid conservation (Fig. 2A). Residues boxed in red indicate strict conservation, while residues boxed in yellow indicate greater than or equal to 80% identity across the 11 homologs. Interestingly, the α-helix structure in proteases of the 1A9M and 1ODX is somewhat longer.

The structural and functional significance of a residue in protein structure is substantial for its evolutionary conservation. The importance of a given residue in conserving the structure and function of a protein can be inferred from the degree of conservation of the residue in a multiple sequence alignment of the protein and its homologues. The ConSurf^31^ is a bioinformatics tool for calculating the conservation pattern of a protein, which quantifies the degree of conservation at each aligned position. This program first identifies conserved positions using multiple sequence alignment, then calculates the evolutionary conservation rate using the empirical Bayesian method and provides the evolutionary conservation profiles of the structure or the sequence of the protein. ConSurf identifies functional regions in proteins, taking into account by considering the evolutionary relationships among their sequence homologs. ConSurf score ranges from 1 to 9, with 1 denoting rapidly evolving (variable) sites, 5 depicting sites that are evolving at an average rate, and 9 representing slowly evolving (evolutionary conserved) sites. The degree of conservation of the amino acid sites among the eleven homologues with similar sequences was estimated (Fig. 2B). Importantly, the information from the sequence logo of the proteins indicates that sequences are highly conserved in different proteins. As expected, the ConSurf analysis has revealed, that most of the amino acids in all the selected proteins are highly conserved. Similarly, this analysis indicated that the functional regions of all proteins are highly conserved.

### Root mean square deviation (RMSD)

**Figure 3:**
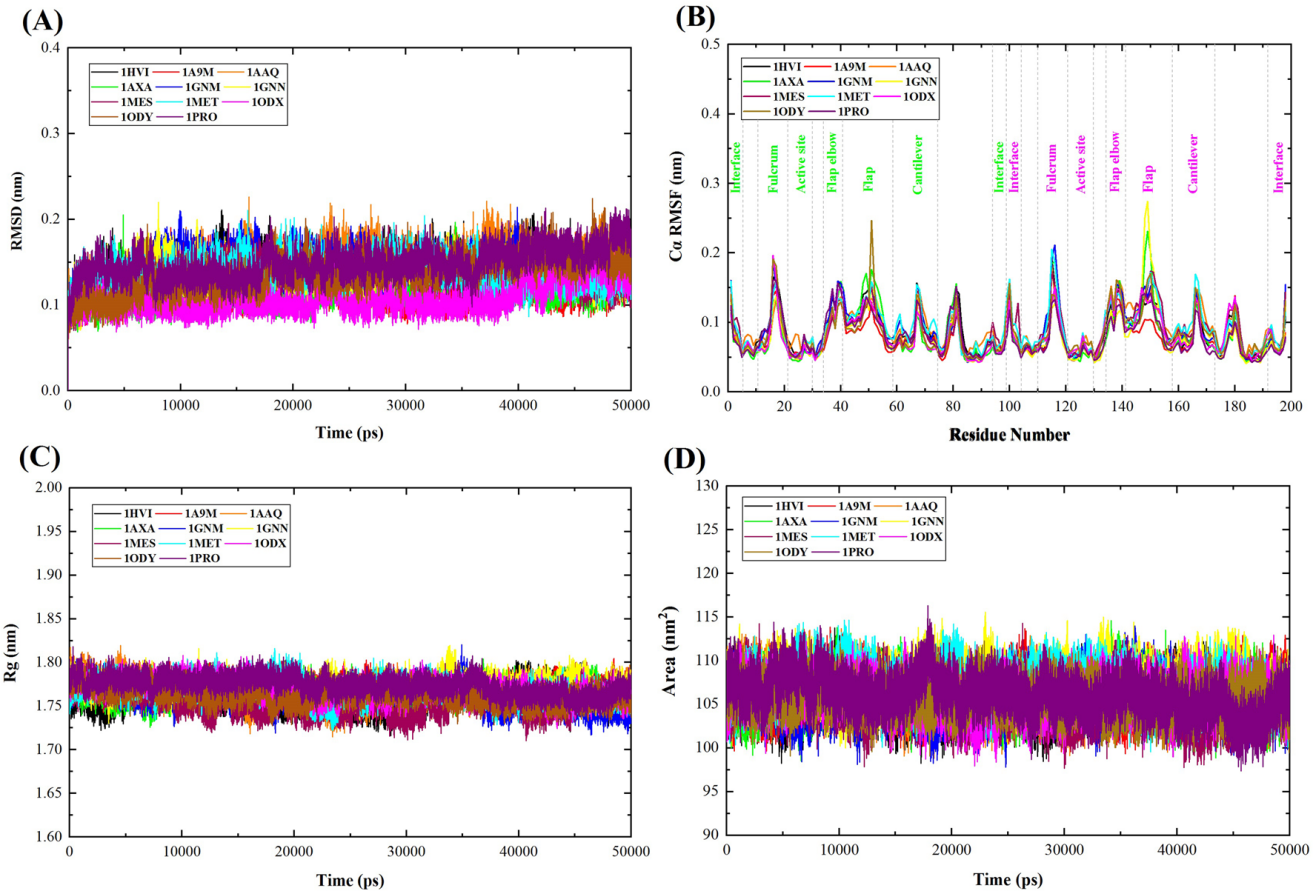
Plot illustrating (**A**) RMSD, (**B**) RMSF, (**C**) Rg, and (**D**) SASA for WT, and mutant proteins. The symbol coding scheme is as follows: wild-type protein (black color), mutant proteins 1A9M (red color), 1AAQ (orange color), 1AXA (green color), 1GNM (blue color), 1GNN (yellow color), 1MES (maroon color), 1MET (cyan color), 1ODX (magenta color), 1ODY (brown color), and 1PRO (violet color).

To obtain information about the conformational stability and assess the reliability of MD simulation, the RMSD of the backbone atoms of all the selected proteins was calculated. The value of RMSD has a negative correlation with the stability of the backbone atoms. The larger the value of RMSD, the more unstable the backbone atoms are^32^. Figure 3A shows the plot of RMSD for 1HVI (native protein) and ten proteins mutated. Initially, in the first 5000 ps, the RMSD was raised due to the "relaxation" of the proteins in the water environment, which is commonly observed in all MD simulation types. It is observed that all the proteins reach equilibrium after ~ 5 ns and present a steady behavior throughout the triplicates and thus suitable for exploring the dynamics of selected proteins.

The RMSD average values for 1HVI, 1A9M, 1AAQ, 1AXA, 1GNM, 1GNN, 1MES, 1MET, 1ODX, 1ODY and 1PRO were found to be 0.146, 0.121, 0.146, 0.115, 0.144, 0.133, 0.133, 0.138, 0.107, 0.135 and 0.148 nm, respectively. 1PRO showed a higher RMSD value as compared to other proteins, whereas 1ODX showed the least value, which confirmed its greater stability than other proteins. The RMSD of wild and mutated proteins did not fluctuate convincingly, and all RMSD average values were less than 0.2 nm, thus, the equilibrium of all MD simulations is reliable. RMSD plots of these proteins showed that they displayed relatively similar stability. The replicates of each protein had slight variations in the RMSD values. Minor deviations in RMSD suggest the stable conformation of all proteins during this time period.

The plateau of RMSD values, observed at all simulations, is approximately similar between the wild-type and mutant proteins, indicating that all structures fluctuate around a stable average conformation. So, it is reasonable to evaluate its local fluctuations^33^.

### Root mean square fluctuation (RMSF)

RMSF was calculated to study better the effect of amino acid mutations on the conformational flexibility of WT and mutant HIV-1 protease variants. The high value of RMSF shows the flexible region, while the low value of RMSF denotes limited movements during MD simulation. A fluctuation value of less than 2 Å is acceptable for a small protein^34^.

The comparison of the fluctuations between WT and mutant structures evidenced that the presence of the mutation resulted in no significant local flexibility alterations (Fig. 3B). The variation in the RMSF values suggested that the fluctuating behaviors were almost similar in the wild-type and mutant proteins except at the flap and fulcrum regions. The RMSF average values for mutant proteins were 0.0762, 0.0904, 0.0817, 0.0887, 0.0804, 0.0886, 0.0919, 0.0816, 0.0848, 0.0797 nm, for 1A9M, 1AAQ, 1AXA, 1GNM, 1GNN, 1MES, 1MET, 1ODX, 1ODY, and 1PRO, respectively, while the RMSF value for WT is 0.0864 nm (Table 1). According to the fluctuation score, we ranked the collected values as follows: 1MET > 1AAQ > 1GNM > 1MES > 1HVI (WT) > 1ODY > 1AXA > 1ODX > 1GNN > 1PRO > 1A9M. Therefore, 1MET showed larger fluctuations as compared to other proteins, whereas 1A9M showed the least fluctuations. In proteins 1A9M and 1ODX, the number of helix pitches in α-helix has increased, as a result, these two proteins have become more stable and show fewer fluctuations.

**Table 1:** The calculated parameters for all the protein obtained after 50 ns MD simulations.

Proteins	Average backbone RMSD (nm)	Average Cα-RMSF (nm)	Average Rg-protein (nm)	Average SASA (nm^2^)
1HVI (WT)	0.146	0.0864	1.759	105.677
1A9M	0.121	0.0762	1.771	106.222
1AAQ	0.146	0.0904	1.766	106.254
1AXA	0.115	0.0817	1.767	105.925
1GNM	0.144	0.0887	1.761	105.518
1GNN	0.133	0.0804	1.772	107.363
1MES	0.133	0.0886	1.754	105.314
1MET	0.138	0.0919	1.768	107.140
1ODX	0.107	0.0816	1.769	105.898
1ODY	0.135	0.0848	1.762	105.717
1PRO	0.148	0.0797	1.773	106.057

The RMSF plot showed that residual fluctuations are present in all proteins in several regions of the structure of the proteins. In addition to the N- and C-terminal residues, the regions around 17(116), 41(140), 52 (151), 67(166), and 81(180) show the biggest dynamic fluctuations. Residues 1–37 and 59–99 in each monomer are defined as the core region, while residues 43–58 constitute the flap region. It is worth noting that for all the proteins, changes observed in one monomer are almost always present also in the other. In the WT and mutant HIV-1 protease variants, there are two very stable regions in both monomers, one in the active site (residues 23–30/122–129) and the other in the α-helix formed by residues 86 to 90. As mentioned, the residues near the catalytic D25/D124 present a high degree of rigidity in all proteins, which is expected, as the catalytic function of these residues probably needs a well-defined stable three-dimensional structure.

Due to the fact that the conformational dynamics of the flap region of the protease are vital for the catalytic activity, our analysis for fluctuation focused on the flap region. Interestingly, after the check of the flap region, which includes residues 43–58, we noticed higher fluctuations in the 1ODY, 1GNN, and 1AXA proteins compared to the WT; while other areas exhibited similar behavior. The handedness feature of the flaps was also visible in the RMSF values in which one of the flaps has more fluctuation than the other^35^. The RMSF results are consistent with that of the RMSD.

**Figure 4:**
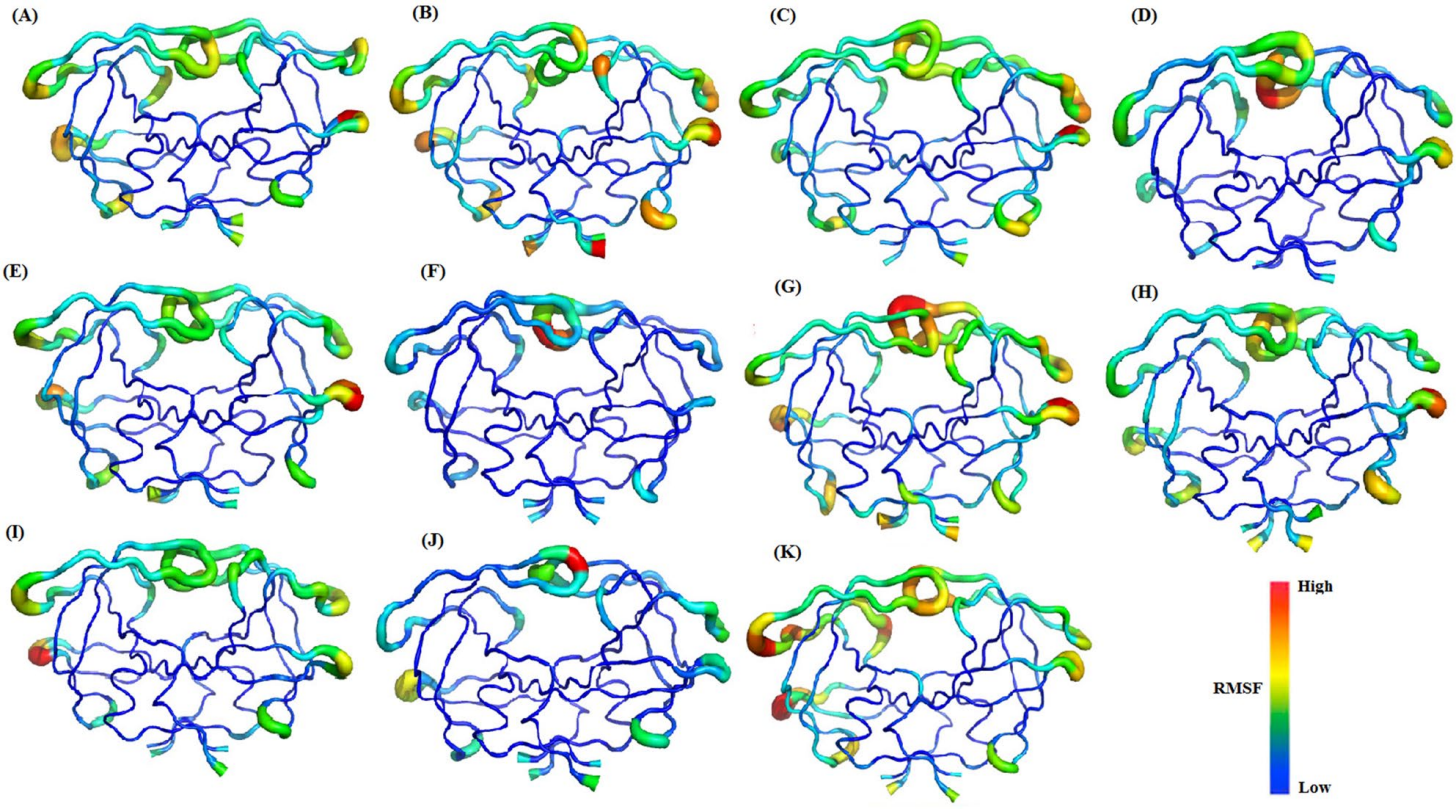
Sausage representation for the selected proteases [(**A**) 1HVI; (**B**) 1A9M; (**C**) 1AAQ; (**D**) 1AXA; (**E**) 1GNM; (**F**) 1GNN; (**G**) 1MET; (**H**) 1MES; (**I**) 1ODX; (**J**) 1ODY; (**K**) 1PRO]. The thickness of the sausage indicates the mobility of the region. The color scale goes from blue (low RMSF values—poorly flexible regions) to red (high RMSF values—very flexible regions). The figures were prepared using the PyMOL software.

To visualize conformational fluctuations in selected proteins, we used a "sausage" plot to show the range of observed motions during simulation trajectories (Fig. 4). The thickness of the sausage plot is proportional to B-factor values and shows the extent of protein chain motion. The thinner segments denote the most stable regions of the protein, while thicker segments represent the more mobile regions. In most of the selected proteases, the highest mobility is found in the flaps and flap elbow regions, which should have significant functional implications because these regions are near the active site.

### Radius of gyration (Rg)

The Rg calculated from the MD trajectory indicates the compactness or rigidity of a protein system during the simulation. Higher Rg values indicate less compactness of protein structure, while lower Rg values indicate more stability and compactness^36^. We performed Rg analysis to observe the conformational alterations and dynamic stability of the wild-type and mutant structures. To understand the changes in Rg with time, a plot was constructed (Fig. 3C). The average values of Rg calculated for each protein can be found in Table 1. The Rg plots of all the protein systems show fluctuations ranging lesser than 2 Å, which shows that the protein systems are stable. The average Rg values for 1HVI, 1A9M, 1AAQ, 1AXA, 1GNM, 1GNN, 1MES, 1MET, 1ODX, 1ODY, and 1PRO were found to be 1.759, 1.771, 1.766, 1.767, 1.761, 1.772, 1.754, 1.768, 1.769, 1.762 and 1.773 nm, respectively (Table 1). According to the fluctuation score, we ranked the collected values as follows: 1PRO > 1GNN > 1A9M > 1ODX > 1MET > 1AXA > 1AAQ > 1ODY > 1GNM > 1HVI (WT) > 1MES. The Rg plot suggested that the 1MES has tight packing than other proteins. Protein 1PRO showed a larger radius of gyration than other proteins, indicating that 1PRO, is less tightly packed. During the simulation, the WT and mutant proteins showed almost a similar pattern in terms of Rg values, indicating there were no important changes in the overall structure and folding of the protein after the mutation. In all of the proteins, the Rg results are in good agreement with that of RMSD and RMSF.

### Solvent-accessible surface area (SASA)

The SASA analysis is used to measure the degree to which an amino acid is exposed to its environment. A higher SASA value denotes a diffused protein structure, while a lower SASA value represents a compact structure. An increase or decrease in SASA value represents a change in the structural conformation of the protein^37^. The SASA values of the WT and ten mutated proteins were analyzed for predicting how the mutations affect the structure of the native protein. The SASA values calculated for the WT and ten mutated proteins with time are shown in Fig. 3D, and average SASA values are depicted in Table 1.

The rank of collected average SASA values are listed as: 1GNN (107.363 nm^2^) > 1MET (107.140 nm^2^) > 1AAQ (106.254 nm^2^) > 1A9M (106.222 nm^2^) > 1PRO (106.057 nm^2^) > 1AXA (105.925 nm^2^) > 1ODX (105.898 nm^2^) > 1ODY (105.717 nm^2^) > 1HVI (105.677 nm^2^) > 1GNM (105.518 nm^2^) > 1MES (105.314 nm^2^). According to the Rg analysis, no significant difference was found between the wild and mutated protein, and a similar effect was also observed in the case of the SASA profile. Thus, the SASA results are also in settlement with the RMSD, RMSF, and Rg results.

### Principal component analysis (PCA)

To gain deeper insight into the large-scale collective motions associated with conformation in the selected proteins, we performed PCA for analyzing the dominant protein conformational patterns in a principal components (PCs) phase space during 50 ns the MD simulations. In fact, we investigated the conformational behavior of the Cα atoms of the proteins by projecting them along the direction of the first three eigenvectors (PC1, PC2, and PC3).

**Figure 5:**
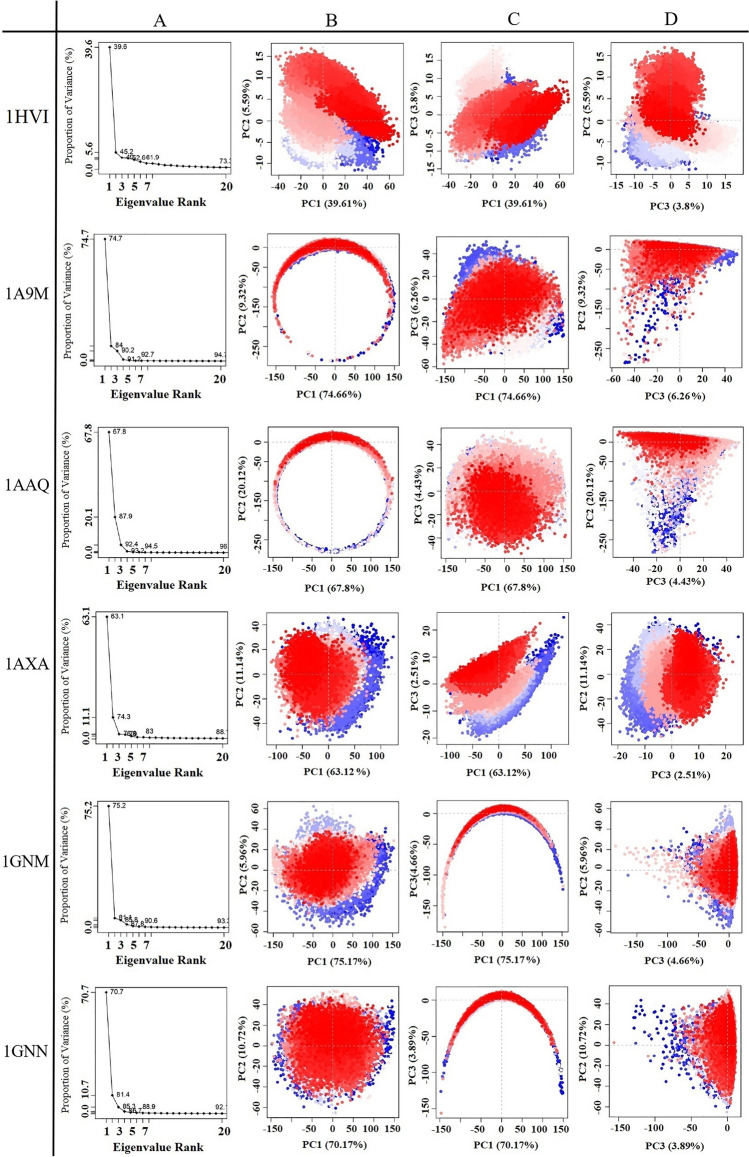
Principal component analysis of the test proteins. This picture shows the proportion of variance (scree) plot (**A**), the projection of PC2 versus PC1 (**B**), PC3 versus PC1 (**C**), and PC2 versus PC3 (**D**) of the WT, 1A9M, 1AAQ, 1AXA, 1GNM and 1GNN, proteins during the simulation period.

**Figure 6:**
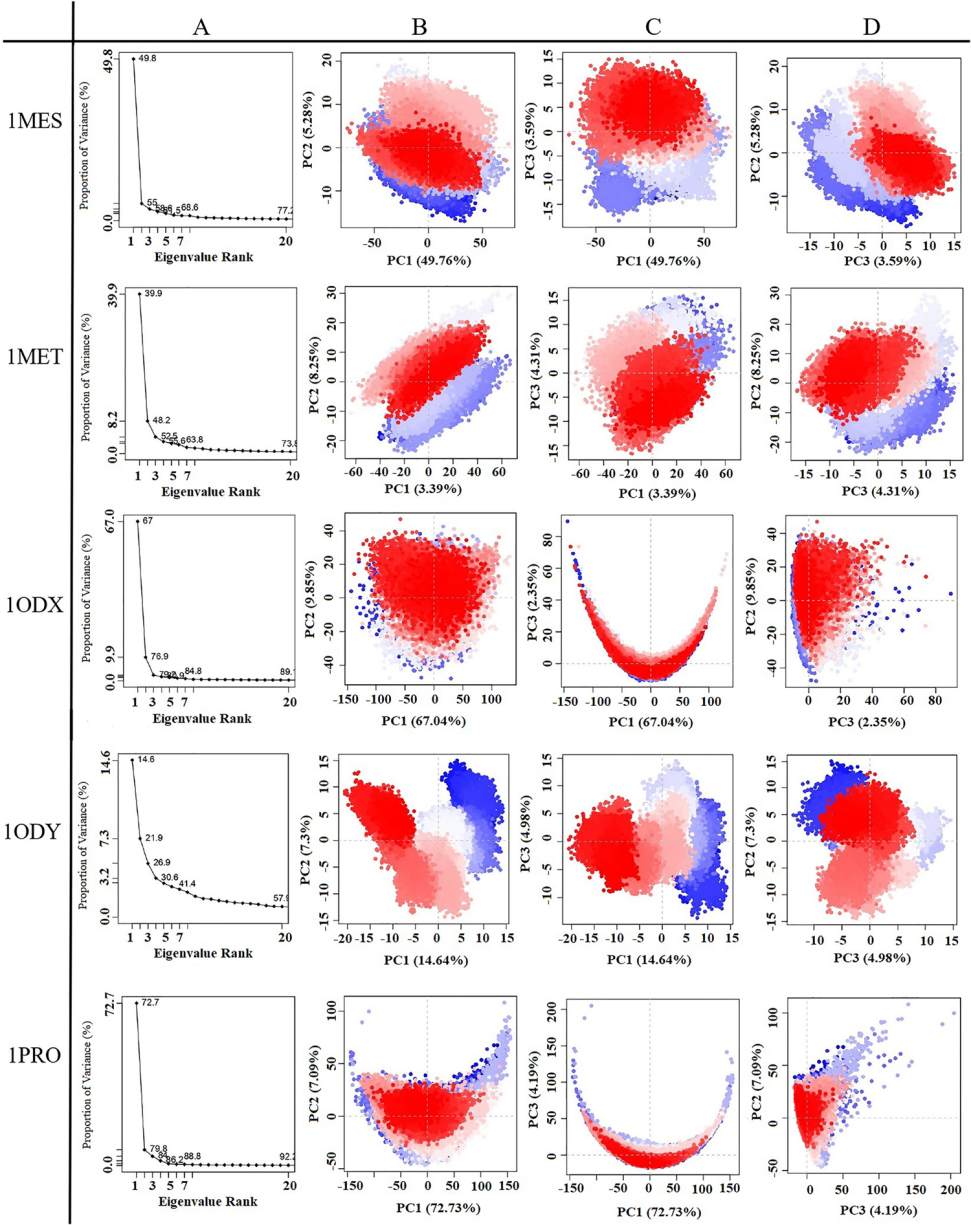
Principal component analysis of the test proteins. This picture shows the proportion of variance (scree) plot (**A**), the projection of PC2 versus PC1 (**B**), PC3 versus PC1 (**C**), and PC2 versus PC3 (**D**) of the 1MES, 1MET, 1ODX, 1ODY, and 1PRO proteins during the simulation period.

Figures 5 and 6 exhibit the first three PCs for selected proteins extracted from respective 50 ns MD simulation trajectories in the form of cluster groups. The 2D principal component plot between eigenvectors 1, 2, and 3 was drawn to compare acceptable conjoined motions. This 2D plot indicates the variations in the ensemble distribution for each conformation during the simulation interval, where each dot represents one conformation of the trajectory at a time *t*. The uninterrupted color representation (from blue to white to red) indicates the presence of substantial periodic bounces between conformers during MD simulations. There were three conformational states in all proteins, including the unstable conformational states (blue dots), the intermediate states (white dots), and the stable conformational states (red dots). These observations supported the compact and cluster motions for all selected proteins in their respective trajectory. Convincingly, dynamic motions of clusters in each extracted PC for the respective protein structure suggested the induction of collective fluctuation by point mutations as a function of the 50 ns MD simulation interval.

Principal components or eigenvectors are ranked according to decreasing eigenvalues, which directly correspond to their contribution to the overall conformational variance. Figures 5A and 6A show the scree plot of eigenvalues for the first 20 PC, which indicates the percentage of the total variance (mean-square fluctuation) captured by each PC based on their eigenvalue rank. In this diagram, tags on each point indicate the cumulative sum of variance accounted that by a specific eigenvector and its previous eigenvectors. Indeed, cumulative variance is shown as a function of the number of PCs. This Figure shows the first few eigenvalues at the beginning are associated with larger concerted motions but decline rapidly and attain more localized fluctuations.

Percent of the variance and cumulative variance for each of the proteins during the 50 ns of MD simulations are presented in Table 2. According to Table 2, PCA showed that the top 20 PCs could capture 73.3%, 94.7%, 96.4%, 88.7%, 93.3%, 92.7%, 77.2%, 73.8%, 89.7%, 57.9% and 92.2% of total variance during the 50 ns of MD simulations in 1HVI, 1A9M, 1AAQ, 1AXA, 1GNM, 1GNN, 1MES, 1MET, 1ODX, 1ODY and 1PRO proteins, respectively. From this result, also it was observed that the first three PCs were 49.0%, 90.2%, 93.2%, 76.7%, 85.7%, 85.3%, 58.6%, 52.4%, 79.2%, 26.9% and 84.0% of the total variance in the motion observed in the trajectories of 1HVI, 1A9M, 1AAQ, 1AXA, 1GNM, 1GNN, 1MES, 1MET, 1ODX, 1ODY and 1PRO, respectively. According to Table 2, PCA suggests that the properties of the motions described by the first three PCs are clearly different for all the proteins. The scree plot of all proteins indicates that the elbow point is located at the third PC, meaning the first three PCs appear to be significant (Figs. 5 and 6). As shown in Figs. 5A and 6A, after the third PC, there are no momentous variations in the eigen fraction till 20 eigenvalues, indicating a state of convergence in the respective proteins. These observations suggested that significant flexibility was produced in all proteins during the initial phase of 50 ns MD simulation which eventually diminished to attain a stable system. Moreover, a steady decrease in the amplitude of an eigen fraction further indicates an additional localized fluctuation in the protein structure to attain a favorable conformation. Comparing all proteins, the highest and lowest PC1 magnitude is 75.1% and 14.6% for 1GNM and 1ODY, respectively. Except for 1ODY and 1MET, the magnitude of PC1 in other proteins is significantly increased, which might correlate with the increased flap movement in the mutant proteins. The mutation in the 1MET protein did not alter the PC1 contribution significantly, but in the 1ODY protein, a lesser PC1 contribution was observed. These observations validated the result of higher flexibility of the mutated proteins compared to the native protein.

**Table 2:** The eigenvalue, percent of the variance and cumulative variance for three principal components of each of the proteins during the 50 ns of MD simulations.

Protein	Principle component (PC)	Eigenvalue	Variance (%)	Cumulative variance (%)
1HVI (WT)	PC1	265.012	39.611	39.611
	PC2	37.407	5.591	45.203
	PC3	25.440	3.802	49.005
1A9M	PC1	2704.054	74.664	74.664
	PC2	337.502	9.319	83.983
	PC3	226.812	6.263	90.246
1AAQ	PC1	4278.441	74.335	74.335
	PC2	831.677	14.450	88.784
	PC3	255.046	4.431	93.216
1AXA	PC1	996.035	63.120	63.120
	PC2	175.818	11.142	74.261
	PC3	39.660	2.513	76.775
1GNM	PC1	2080.934	75.166	75.166
	PC2	164.879	5.956	81.121
	PC3	128.989	4.659	85.781
1GNN	PC1	1780.544	70.707	70.707
	PC2	269.991	10.722	81.428
	PC3	98.061	3.894	85.322
1MES	PC1	413.644	49.757	49.757
	PC2	43.914	5.282	55.040
	PC3	29.853	3.591	58.631
1MET	PC1	297.357	39.901	39.901
	PC2	61.484	8.250	48.151
	PC3	32.091	4.306	52.457
1ODX	PC1	1104.702	67.038	67.038
	PC2	162.356	9.852	76.890
	PC3	38.706	2.349	79.239
1ODY	PC1	50.291	14.642	14.642
	PC2	25.083	7.303	21.946
	PC3	17.118	4.984	26.929
1PRO	PC1	1766.330	72.730	72.730
	PC2	172.274	7.094	79.824
	PC3	101.688	4.187	84.011

Except for 1ODY, the PC1 accounts for more than one-third of the total variance and strongly overcomes the total variance showing the global dynamics^38^. Interestingly, all the selected proteins, except 1ODY, showed a sharp increase in the percentage of variance corresponding to the first three PCs and covered more than > 50% of the total proportion of variance of atom positional fluctuations in each simulated protein. After that, the individual component contributions fall below 3%. In fact, these first three PCs account for a large proportion of the overall protein conformation and capture the most significant dominant motions, in other words, the fluctuations of the highest amplitude that are generally biologically relevant motions. These results showed that point mutations caused significant changes in the conformational motions of the selected proteins. Therefore, PCA dots images generated from the first three eigenvectors are used to observe the conformational transitions of these proteins^39,40^.

On a 2D principal component plot, the larger the cumulative variance on the two considered principal components, the more significant the distance between the points. It means that diverse conformations will have diverged while similar conformations will have grouped on the 2D PCA plot^38^. Comparing the 2D scatter plots of all proteins, it could be seen that the conformational states of the mutated proteins compared with wild protein had changed significantly. As shown in Figs. 5 and 6, except 1ODY, the PC2 versus PC1 plots for all proteins clearly indicated the conformers visit a large conformational space.

Evaluation of internal motions through the first three principal components shows that PC1 and PC2 are prominently related. As shown in Figs. 5 and 6, the PC2 versus PC1 plots obtained from the MD trajectories are almost varied for all proteins, which display differences in motion across the two first principal components. These observations clearly indicated the differences in protein motion and the conformational landscape between the proteins. This indicates that mutations in these proteins have caused conformational changes. However, the presence of overlap between blue and red colors indicates that the protein does revisit the same state during the simulation, even though it undergoes conformational changes. Also is an indication of the quality of sampling, which illustrates the simulation time is sufficient. As shown in PC2 versus PC1 plots, in the case of comparing proteins with each other, the 1ODY protein has a smaller phase space and lower performance flexibility than other proteins. In the 1ODY protein, the contribution of PC1 and PC2 to the variance is 14.6% and 7.3%, respectively, while other PCs contributed no more than 5.0%.

It can be found from Figs. 5 and 6 that in PC2 versus PC1 diagrams, the points of all proteins are almost evenly distributed near the midline, while in PC2 versus PC3 diagrams, the points of proteins are differently distributed. The closer the point distribution in Figs. 5 and 6 indicates that the protein system is more stable, so the 1ODX and 1AXA proteins are in a more stable state. In another word, 1ODX and 1AXA proteins exhibited the most favorable converged conformations and limited variation against other proteins during MD simulation; suggesting considerable stability as noted from the respective RSMD and RMSF values.

In some PC2 versus PC1 and PC3 versus PC1 diagrams, the projection of the points of proteins reveals a semicircle or U-shape pattern. This type of pattern has been attributed to random diffusion of motion in proteins, allowing only to inform on more accessible degrees-of-freedom for thermal motion along our studied time scale^41^.

Point mutations increase overall collective motions in selected proteins because these mutations effectively increase the Cα movements of proteins. In fact, these mutations have increased the flexibility of proteins. Except for 1ODY, the conformational space covered in other proteins proved to be broader than that of 1HVI. These results mean that after point mutation, the dynamics of the mutated proteins change, which may be required for proper protein function. Also, these fluctuations registered in each protein may be regarded as a requirement for the stability of the relevant protein during MD simulation as a function of time^41^. The PCA results are consistent with the RMSD, RMSF, Rg, and SASA results.

### Porcupine plot

The main motions of protein residues can be better observed and analyzed by displaying eigenvectors as porcupine plots^42^. Porcupine plots are drawn using the custom-made program PyMOL^43^ to visualize the movements of the first three PCs obtained from the principal component analysis. It corresponds to an outline of protein motion, suggesting what part of the protein moves in concert and in which direction. In fact, the first and last eigenvectors from any PC were generated using the PyMOL tool and presented as a porcupine plot. The extent and direction of the most dominant motions of all proteins were visualized through porcupine plots using the ‘modevectors.py’ script (written by Sean M. Law) in PyMOL version 1.7. The plot shows a cone for each Cα atom reflecting the direction of its motion, where the length of the cone indicates the motion amplitude and the size of the cone specifies the number of such Cα atoms. The linear interpolations between the first and last eigenvectors are shown with the color transition from blue to red to highlight the conformational differences between them.

**Figure 7:**
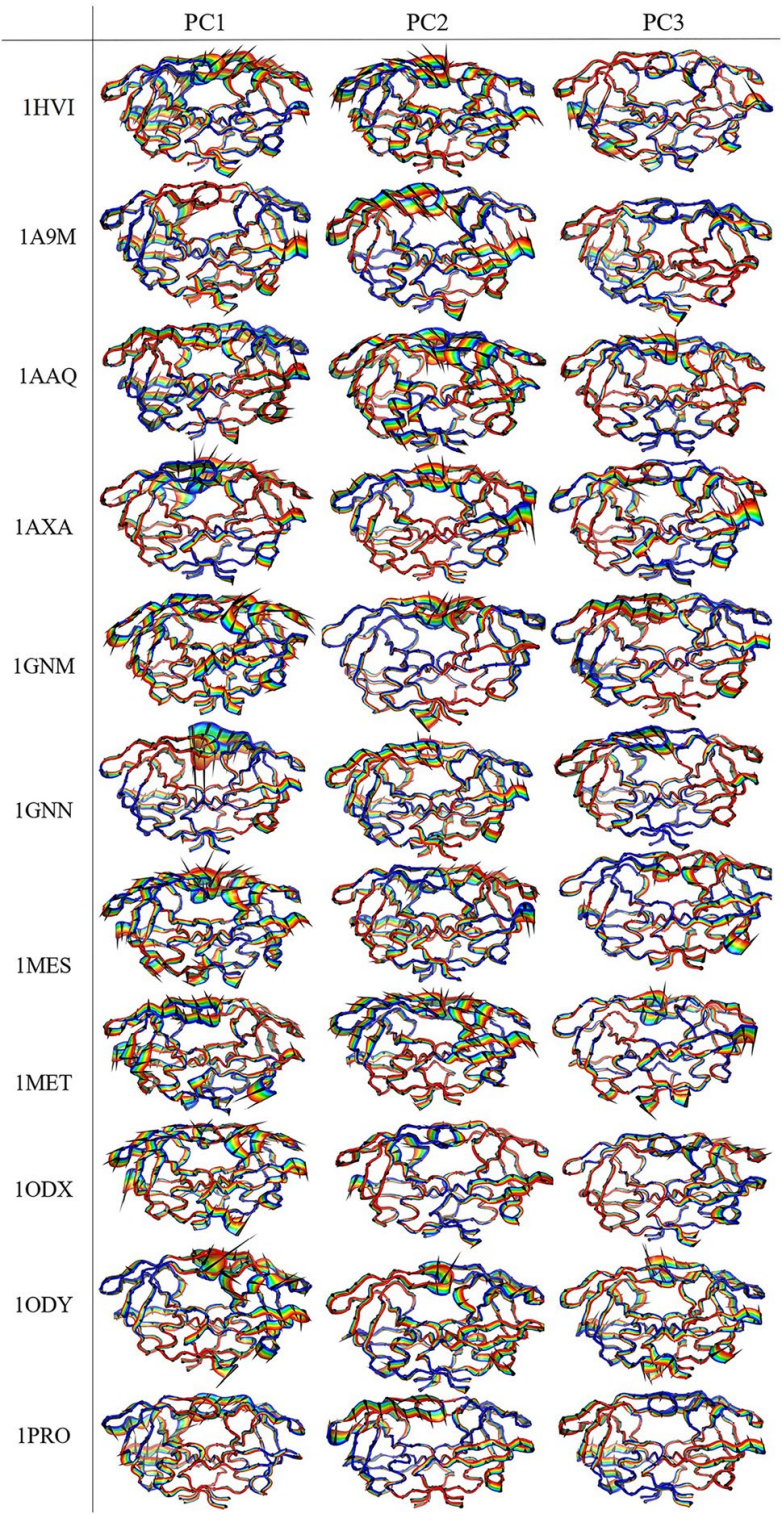
Porcupine plots corresponding to PC1, PC2, and PC3 obtained from PCA on the MD trajectories of the wild-type protein and the mutant proteins. The cones in black represent the direction of the concerted motion, and the length of the cones represents the magnitude of the movement.

The porcupine plots showing the motions of all selected proteins, along the directions of PC1, PC2, and PC3 are presented in Fig. 7. The cones in black represent the direction of the concerted motion, and the length of the cones represents the extent of the motion. Based on the porcupine plots, it is evident that point mutation increases the overall movements of all mutant proteins as compared to the wild-type protein. All proteins showed anti-symmetric movement patterns in the flap, flap elbow, fulcrum, and cantilever regions. In all proteins, the flap and flap elbow regions experienced more flexibility compared to the different segments of the protease, as indicated by the length of the vectors. Thus, it is obvious that in all proteases the flaps and flap elbow motions are highly correlated. Flexible regions of the proteins are not simply the result of loose packing or instability but have been evolutionarily selected^44^**.** Considering that the conformational flexibility of the flap region is necessary for the activity of the HIV-1 protease, it can be concluded that the flap regions are one of the most important signs of the evolution of conformational dynamics in HIV-1 protease.

### Dynamical cross-correlated map (DCCM) analysis

**Figure 8:**
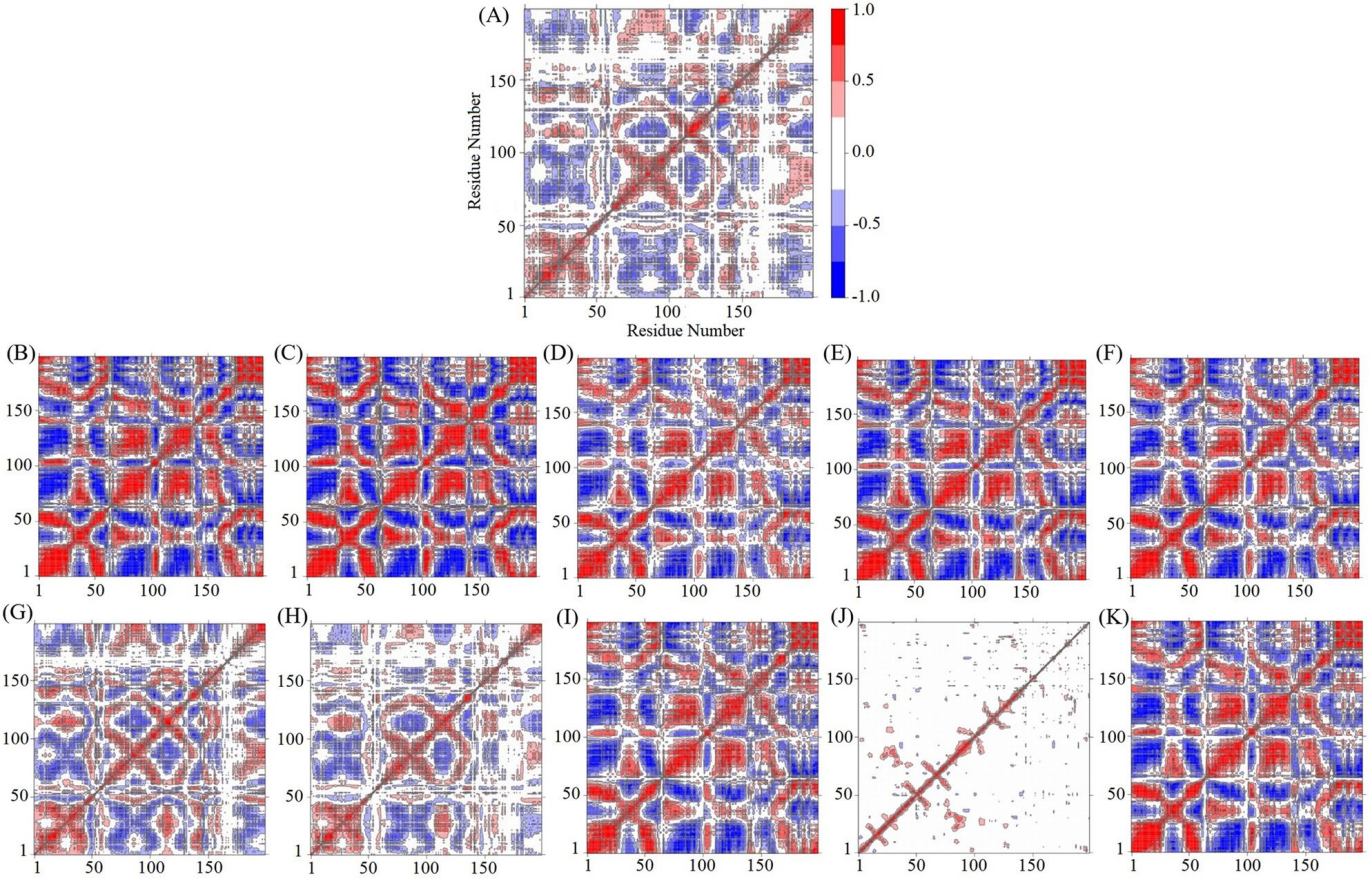
Comparison of cross-correlation matrices of wild-type and mutant proteins. Cross correlation matrix of C-alpha atoms during 50 ns simulation for wild-type protein (**A**) and mutant proteins of 1A9M (**B**), 1AAQ (**C**), 1AXA (**D**), 1GNM (**E**), 1GNN (**F**), 1MES (**G**), 1MET (**H**), 1ODX (**I**), 1ODY (**J**), and 1PRO (**K**). The range of motion is shown by different colors in the panel. Red color denotes positive correlation whiles blue color denotes anti-correlation.

To investigate further the effects of point mutation on the conformational dynamics, DCCM analysis was used for Cα atom fluctuations during the 50-ns MD simulation for analyzing the correlation motions of each protein. The cross-correlation analysis could show the relationships between residues and between various regions by quantifying their relative motions. The correlation was normalized and varied from − 1.0 and + 1.0 (from dark blue to white to red) (Fig. 8). The positive correlation (red regions) represented residues moved in the same direction and the negative correlation (blue regions) meant residues moved in the opposite direction^45^. The deeper color indicated a stronger positive correlation or negative correlation. The white regions ranging from − 0.25 to 0.25 were considered as a low correction. A diagonal point represents the Cα atom of the same residue along both axes; therefore, the diagonal elements show the maximum correlation.

The cross-correlation matrix of the C-alpha displacement indicated complex correlated and anti-correlated motions in the wild-type protein and all mutant proteins except 1ODY. The diagonal parts show obvious correlated movements. In the cross-correlation matrix, the *C*_*i*,*j*_ elements of the matrix were found to be symmetrical about the diagonal. Interestingly, in all mutant proteins, correlated and anti-correlated motions increase. The only exception is the 1ODY mutant in which correlated and anti-correlated motions highly decrease. For the mutated proteins, we found deeper shades of red and blue, distinguishing regions of high correlation and anti-correlation that corroborate the fact that mostly global motions are seen for these proteins. In most mutant proteins, except 1ODY, almost all residues have concerted motions and they move in an anti-correlated fashion with the rest of the structure (Fig. 8). The point mutation has significantly changed the direction and the cooperativity of motion in 1ODY.

It can also be observed that the motion of atoms in all mutant proteins as compared to the wild-type protein is more compact, while the 1ODY mutated protein represented a very deformed form of the backbone atomic motion. The 1MES and 1MET proteins have approximately the same correlation and anti-correlation motions as the 1HVI protein, however, in these two mutant proteins, the intensity of the correlated and anti-correlation motions is slightly higher than that of the 1HVI protein. Thus, our results show that variations in sequence can lead to changes in dynamics without altering structure.

As seen from principal components and the dynamic cross-correlation maps, the point mutations change the major motions of the proteins and may improve the dynamic behavior of proteins. These results indicate that point mutations may have a pronounced effect on the conformational flexibility of proteins.

The conformational dynamics in the flaps of HIV-1 protease plays a critical role in the mechanism of substrate or inhibitor binding. Opening of the flaps is essential for the entrance of substrate into the binding cleft and flaps in the closed conformation sturdy the substrate for catalysis. The protease dimer is in dynamic equilibrium between the closed conformation and different open conformational states. These conformational changes are highly associated with the flexibility of the flaps of the protease^46^. Molecular dynamics studies show that mutations in the flap regions may affect the dynamics of the flap and thus the binding of protease inhibitors^47^. The results revealed that the mutations caused increased movement in the flap, and flap elbow regions. The presence of a region with such conformational dynamic diversity in the protein that also provides the necessary flexibility to perform the proper function of the protein indicates that the protein has also evolved dynamically in addition to structural evolution. Indeed, since the flexible regions of proteins are selected evolutionarily, it can be inferred that the flap regions are the most significant sign of the evolution of conformational dynamics in the HIV-1 protease.

Previous studies show that dynamic structural regions exist in various proteins whose conformational flexibility is essential for the function and allostery of proteins (Table 3). Thus, it is time to group these dynamic structural regions and choose a specific and unique name for these areas. Our suggestion for naming these regions is dynamozones. Dynamozones are dynamic structural regions in some proteins that contribute to the biological function and allostery of proteins via their convenient flexibility. These regions provide the flexibility needed for proteins to suitable function, and their presence is completely essential for protein function. One of the characteristics of these regions in most proteins is the presence of the amino acid glycine in their structure. Dynamozones are a subset of three main groups: loops, hinges, and linkers. One of the clear signs of the evolution of conformational dynamics of proteins is the presence of dynamozones in proteins because these dynamic regions have evolved in such a way that they workable the function and allostery of proteins. These regions via different mechanisms such as performing opening-closing movements for ligand binding to the active site, appropriate motions of the loops for placement of the catalytic residues in the suitable position for catalysis, switching from an inactive "out" conformation to an active "in" conformation to create the catalytically active form of the enzyme, proper motions of linkers in proteins to accept compact and extended conformations, movements of hinges to connect antibodies to antigens, etc. help various proteins to perform their proper function. Thus, we can say that dynamozones are a footmark of the evolution of the conformational dynamics of proteins. The details of some of the know dynamozone members are listed in Table 3.

**Table 3:** List of some known members of Dynamozone.

Structure	Dynamozones	Proteins	Role or Function	References
Loop	Flap	• HIV protease • Plasmepsins • Beta-secretase • Cathepsin • Pepsins	These regions control the entrance and stabilization of ligands in the active site	^67–70^
Loop	Loop (Residues 166–176)	• Triosephosphate isomerase	In the ligand-bound state, the loop moves for ∼7 Å as a rigid lid toward the active site and accepts a “closed” conformation. These motions of the rigid lid close to the active site are essential for the catalytic mechanism of the enzyme	^71–73^
Loop	Loop	• Enolases • Aldolases	Movements of the loop permit the catalytic residues to be oriented in a suitable position for catalysis	^74–76^
Loop	WPD loop	• Protein tyrosine phosphatases (PTPs)	This loop closes over the active site upon binding of the substrate, and loop closure permits the correct orientation of catalytic residues around the ligand	^77,78^
Loop	Met20 loop (Active site loop (	• Dihydrofolate reductase (DHFR)	This loop acts as a lid that closes on the cofactor, thereby allowing DHFR to adopt occluded and closed conformations	^79–81^
Loop	Helical loop	• Lipases	This loop is important for the enzyme function, acting as a lid to open or close the hydrophobic active site	^82,83^
Loop	Long loop	• β1,4-galactosyl transferase	A displacement of more than 20 Å this long loop in protein provides binding sites for various ligands	^84^
Loop	Omega loop	• Cdc34-like E2 enzymes	This loop can act as a lid that regulates the accessibility of the catalytic site and disturbs the charging activity of ubiquitin until a conformational change toward an open state is promoted by phosphorylation	^85–87^
Linker	Flexible linker	• Calmodulin (CaM)	CaM has two globular domains connected by a short and flexible linker that permits the protein to accept a wide variety of extended and compact conformations	^88–90^
Hinge Loop Linker	• Hinge region • P-loop • hydrophobic “spines” • A-loop • αC helix • DFG motif • αB helix	• Protein kinases (PKs) • Tyrosin kinase • Src protein kinase	Hinge region: The hinge motion is necessary for the opening and closure of the kinase catalytic domain (CD) P-loop (β1-β2 loop or G-loop or Gly-rich loop): This flexible loop is very important for the coordination of ATP phosphates Hydrophobic “spines”: Two hydrophobic “spines” link the two lobes of protein kinase and dynamically connect all the elements important for catalysis A-loop (Activation loop): In the inactive state of the enzyme, the A-loop is folded onto itself, and its opening is required to create the catalytically active form αC helix: This helix in the “in” active conformation forms a hydrogen bond with the β3 strand for creating the catalytically active form of the enzyme DFG motif: This motif in the active site switches from an inactive (DFG-out) conformation to an active (DFG-in) conformation, which is necessary to create the catalytically active form of the enzyme αB helix: This helix creates a cavity, the so-called PIF pocket, which is very important for allosteric regulation of the protein kinases belonging to the AGC family	^91–96^
Hinge	Hinge region	• Lactoferrin	The hinge motions permit the formation of the complete iron-binding site in the closed states of lactoferrin	^97–100^
Hinge	Hinge region	• Immunoglobulins	The hinge region is connecting the Fab (Fragment antigen binding) region to the Fc (Fragment crystallizable) region	^101,102^

## Conclusions

Protein dynamics, structure, and function are highly correlated. In the case of the HIV-1 protease, the dynamics of conformational changes are very critical for enzyme function. In the present study, we have explored the effect of mutations on the dynamic behavior of the WT and mutant HIV-1 proteases variants using a combination of MD simulations, cross-correlation analysis, and PCA. Sequence similarities between proteins were strikingly similar and confirmed the assumption that proteins with greater sequence similarity behave similarly. The results indicate that mutations not only produce important effects on the correlated motions and flexibility of HIV-1 protease but also increase the stability of HIV-1 protease during the simulation period. These positively selected mutations introduce significant flexibility in important regions such as the flap and flap elbow. Further, the parameters such as RMSF, DCCM, porcupine plot, and the PCA analysis revealed increased fluctuation/motion in the mutated proteases in comparison to the wild-type structure. Our main results were as follows: (1) The flap regions are the most evident indication of the evolution of conformational dynamics in HIV-1 protease and are an excellent case for investigating the evolution of conformational dynamics; (2) Dynamozones are dynamic structural regions in some proteins that donate to the biological function and allostery of proteins through their proper flexibility; (3) Because of the presence of other members of dynamozones in various proteins, we offer to consider dynamozones as an indication of the evolution of the conformational dynamics of proteins. The flap regions are one of the most significant dynamozone members that are crucial for HIV-1 protease function.

## Computational methods

### Protein structures preparation

A set of eleven HIV-1 protease proteins with experimentally determined structures chosen from the Protein Data Bank (PDB), were used in this study^48^. The selected proteins' structures have been determined using X-ray crystallography, and their resolution and R-factor are less than 3.0 and 0.19, respectively. Molecular dynamics simulation approaches pave the way for an in-depth analysis of the effects of mutations on protein structure and dynamics. In the present study, mutations G48H, L63I, A28S, V82D, V82N, (I3V; I84V), (I3V; V82F), (A71T; V82A), (I3V; R41A) and S37N, which are related to structures 1A9M, 1AAQ, 1AXA, 1GNM, 1GNN, 1MES, 1MET, 1ODX, 1ODY and 1PRO, respectively, were analyzed to investigate the effect of mutations on the conformational dynamics of HIV-1 protease using molecular dynamics simulations (Fig. 1B). The PDB code: 1HVI was also selected as a wild-type protein. We selected a suitable mutation from each of the different parts of the HIV-1 protease to study the dynamic behavior of the proteases. The ligands, ions and water molecules were removed from the protein structures. The details about the selected HIV-1 protease variants are listed in Table 4.

**Table 4:** Overview of the amino acid changes in the HIV-1 protease families.

Simulated proteins (PDB ID)	Residue position	Amino acid change	Property change
1HVI (WT)			
1A9M	48	G → H	Hydrophilic/+ *
1AAQ	63	L → I	Hydrophobic/Hydrophobic
1AXA	28	A → S	Hydrophobic/Hydrophilic
1GNM	82	V → D	Hydrophobic/−**
1GNN	82	V → N	Hydrophobic/Hydrophilic
1MES	3	I → V	Hydrophobic/Hydrophobic
	84	I → V	Hydrophobic/Hydrophobic
1MET	3	I → V	Hydrophobic/Hydrophobic
	82	V → F	Hydrophobic/Hydrophobic
1ODX	71	A → T	Hydrophobic/Hydrophilic
	82	V → A	Hydrophobic/Hydrophobic
1ODY	3	I → V	Hydrophobic/Hydrophobic
	41	R → A	+ /Hydrophobic
1PRO	37	S → N	Hydrophilic/Hydrophilic

* + Positively charged amino acids; **− negatively charged amino acids.

### Molecular dynamics simulation

In this study, all of the MD simulations were performed using the GROMACS 2016.3^49^. The force field parameters were assigned according to the Amber99SB*-ILDN force field^50^. Protonation states were assigned to each structure using PDB2PQR^51^ through ProPKa^52^ at pH 7.0. The TIP3P water model^53^ was used to solvate the system, which was generated as a cubic box like area with a side of 1 nm such that the protein is covered appropriately with water molecules. All proteins were electrostatically neutralized by adding chlorine ions around the molecules. Each protein was minimized by the steepest descent algorithm up to a maximum of 50,000 steps and a convergence tolerance of 10 kJ mol^−1^ nm^−1^. The energy-minimized structure of the native protein and ten mutant proteins were used as the starting points for the MD simulations. Equilibration of the proteins was conducted in two phases NVT and NPT ensembles each for 100 ps. Particle-Mesh-Ewald (PME) method^54^ with a 1.0 nm cutoff was used to calculate the electrostatic interactions. During the MD simulation, the LINCS algorithm^55^ was used to constrain all the bonds. The temperature was kept constant (300 K) and pressure was maintained at 1 bar using the V-rescale thermostat^56^ and the Parrinello-Rahman barostat^57^, respectively. Following the equilibrium methods, MD simulations were performed for each of the native and mutant proteins with 3 repetitions for 50 ns.

### Analysis of trajectory files

The resulting trajectory files of the simulations were studied employing different parameters existing in GROMACS utilities. From the refined trajectories, various parameters, such as root mean square deviation (RMSD), root mean square fluctuation (RMSF), the radius of gyration (Rg), and solvent-accessible surface area (SASA) for all proteins were calculated. The sequence alignments were created with T-Coffee^58^ and ESPript^59^. PCA was carried out using the Bio3D package^60^ and used to reveal the changes in the motion patterns of the protein systems.

To exclude the possibility of stochasticity and to confirm the first simulation performed, all 11 selected structures were simulated with different initial velocities with three repeats for 50 ns.

PCA analysis which is explained in detail below is one of the main analyzes performed for this research. To confirm that our simulation time is sufficient to investigate the overall dynamic behavior of the protein, we performed the cosine content analysis, which is part of the PCA method. As a result of this analysis, we can determine whether the protein exhibits random diffusion dynamics during the simulation. A cosine content close to 1 indicates random motion in the protein and non-convergence of the simulation time in the selected time interval, so it cannot be considered for PCA analysis. It has been reported that the cosine content close to 0.2 and sometimes up to 0.5 indicates the non-random diffusion dynamics of the protein during the simulation time and is reliable for further analysis, such as the free energy landscape (FEL)^61,62^.

In this study, we reported the replicates that had cosine content values of the first two eigenvectors (PC1 and PC2) close to 0. Based on chosen principal components (PC1 and PC2), we generated the FEL to calculate Gibbs's free energy value for selected structures. The cosine content values, Gibbs’s free energy values, and RMSD of the simulated structures are reported in the supplementary data file (Figures S1-S11, Table S1).

### Principal component analysis

A principal component analysis was performed to investigate conformational flexibility and the collective motions of the selected proteins using the Bio3D package installed in the R program. This method is based on diagonalization of the covariance matrix of atomic fluctuations to obtain orthogonal eigenvectors and the corresponding eigenvalues. The eigenvectors are the principal components that represent the directions of the coordinated motions of atoms. The eigenvalues indicate the magnitude of the motions along the movement direction^63^. The ensemble formula used to obtain a covariance matrix with elements *C*_*ij*_ for coordinates *i* and *j* is given as:

1 $$C_{ij} = \left\langle {\left( {x_{i} - \left\langle {x_{i} } \right\rangle } \right)\left( {x_{j} - \left\langle {x_{j} } \right\rangle } \right)} \right\rangle \quad \left( {i,j = {1},{2},{3}, \ldots ,{3}N} \right)$$

where *x*_*i*_ and *x*_*j*_ are the mass-weighted Cartesian coordinates of the *i*th and *j*th Cα atoms, *N* is the number of the Cα atoms considered, and $\left\langle {x_{i} } \right\rangle$ and $\left\langle {x_{j} } \right\rangle$ represent the time average over all the configurations obtained in MD simulation. In this study, by the Bio3D package, Cα atoms from 50,000 frames obtained through 50 ns trajectory were superimposed on the initial pose to minimize the root mean square variations between the equivalent residues using fit.xyz function^64^.

### Dynamic cross-correlation map analysis

The cross-correlation analysis can provide information about the impact of mutations on protein dynamics by analyzing how atomic displacements were coupled^65^. The extent of correlative motion of two atoms (or two residues) can be denoted by the cross-correlation coefficient, C_ij_. It is defined by:

2 $$C_{ij} = \frac{{\left\langle {\Delta x_{i} \cdot \Delta x_{j} } \right\rangle }}{{\left( {\left\langle {\Delta x_{i} } \right\rangle^{2} \left\langle {\Delta x_{j} } \right\rangle^{2} } \right)^{1/2} }}\quad \left( {i,j = 1,2, \ldots 3N} \right)$$

where *i* (*j*) means *i*th (*j*th) residue (or atom), Δ*xi* and Δ*xj* are the displacements from the mean position of *i*-th and *j*-th residues (or atoms), with respect to time, respectively, and *N* represents the number of Cα atoms. The angular brackets “〈 〉” illustrate the time average on the whole trajectory. The value of the cross-correlation coefficient is from − 1 to + 1. The positive value implies positively correlated movement (moving in the same direction), and the negative value implies anti-correlated movement (moving in the opposite direction). Higher values of the absolute value of C_ij_ show two residues (or atoms) are more correlated (or anti-correlated)^66^. In this article, we calculated the cross-correlations for all Cα atomic fluctuations extracted from the MD trajectory by using the Bio3D packages of R.

## Data availability

The datasets generated during and/or analysed during the current study are available from the corresponding author on reasonable request.

### Supplementary Information

Supplementary Information.
