# Supplementary material for: Dynamozones are the most obvious sign of the evolution of conformational dynamics in HIV-1 protease
Source: Sci Rep. 2023 Aug 30;13:14179. doi: 10.1038/s41598-023-40818-x (PMC10469195; doi:10.1038/s41598-023-40818-x)
Supplement: Supplementary file 1 — Supplementary Information. [file 41598_2023_40818_MOESM1_ESM.docx]

***Dynamozones* are the most obvious sign of the evolution of conformational dynamics in HIV-1 protease**

Mohammad Rahimi**^1^**, Majid Taghdir**^1^**^,^ *, Farzane Abasi Joozdani**^1^**

**^1^**Department of Biophysics, Faculty of Biological Science, Tarbiat Modares University, Tehran, Iran, 14115_111

*Corresponding author: Majid Taghdir, E-mail address: [Taghdir@modares.ac.ir](mailto:Taghdir@modares.ac.ir)


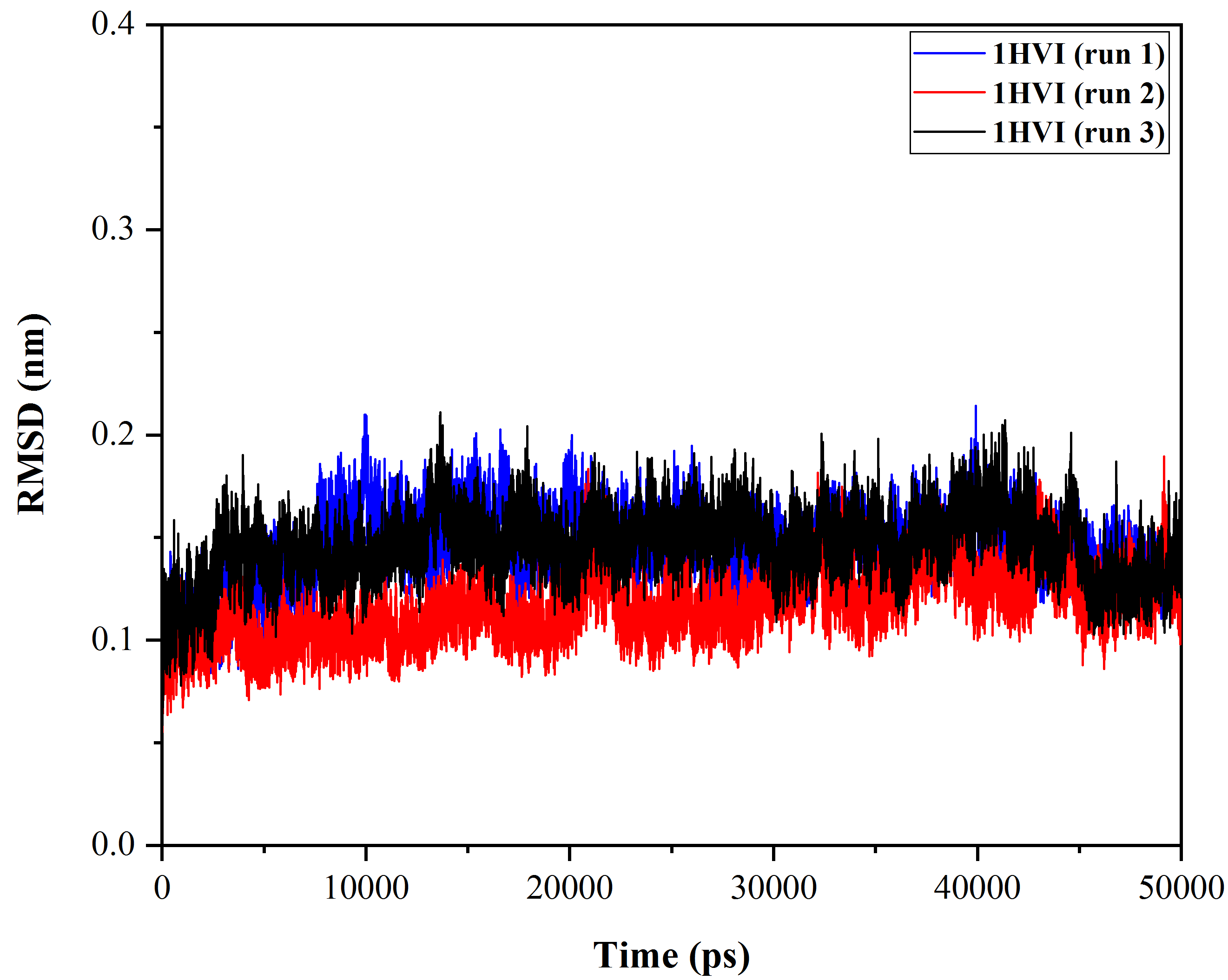


**Figure S1.** RMSD for HIV-1 protease protein backbone atoms (1HV1 code) for 3 repetitions of molecular dynamics simulations.


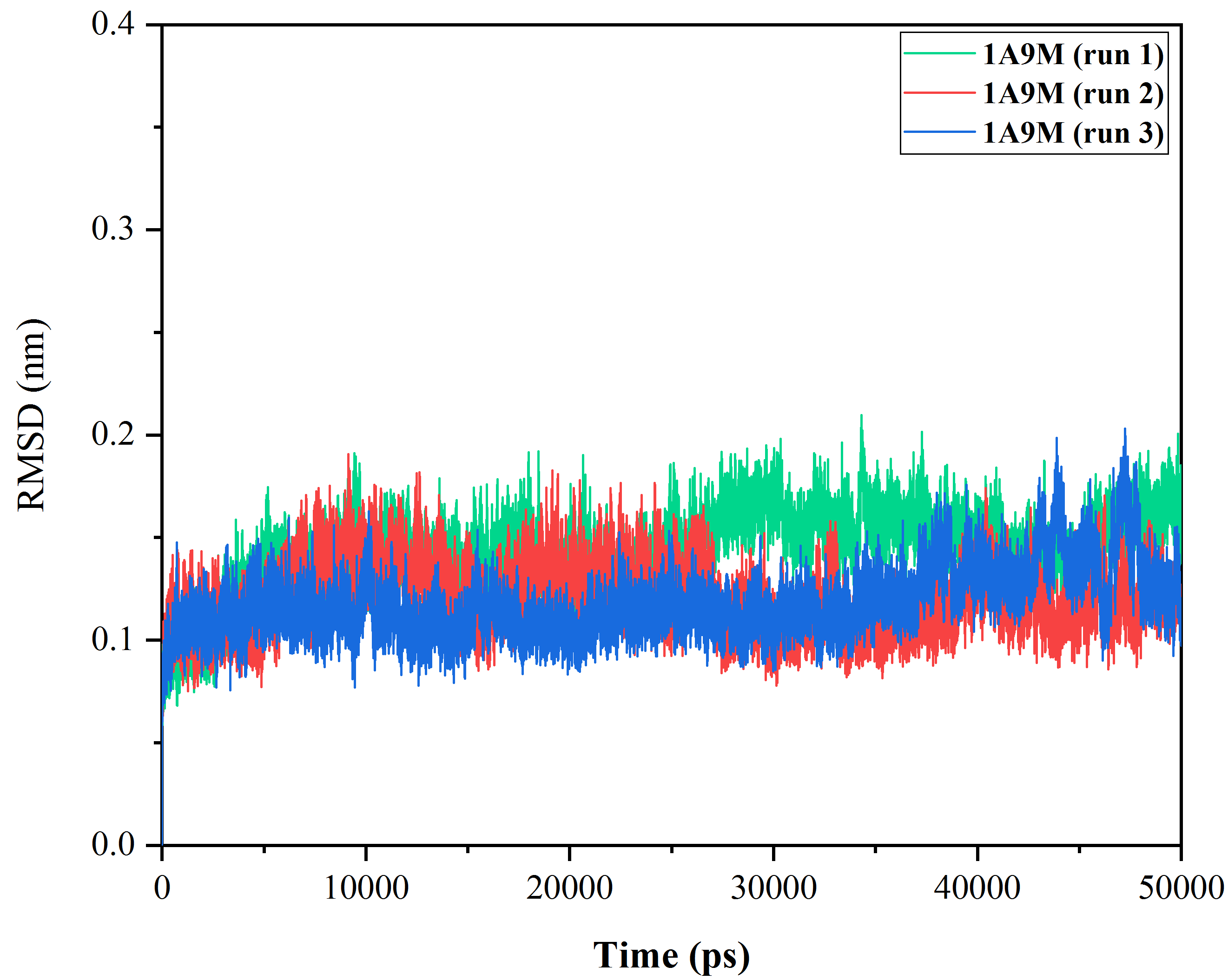


**Figure S2.** RMSD for HIV-1 protease protein backbone atoms (1A9M code) for 3 repetitions of molecular dynamics simulations.


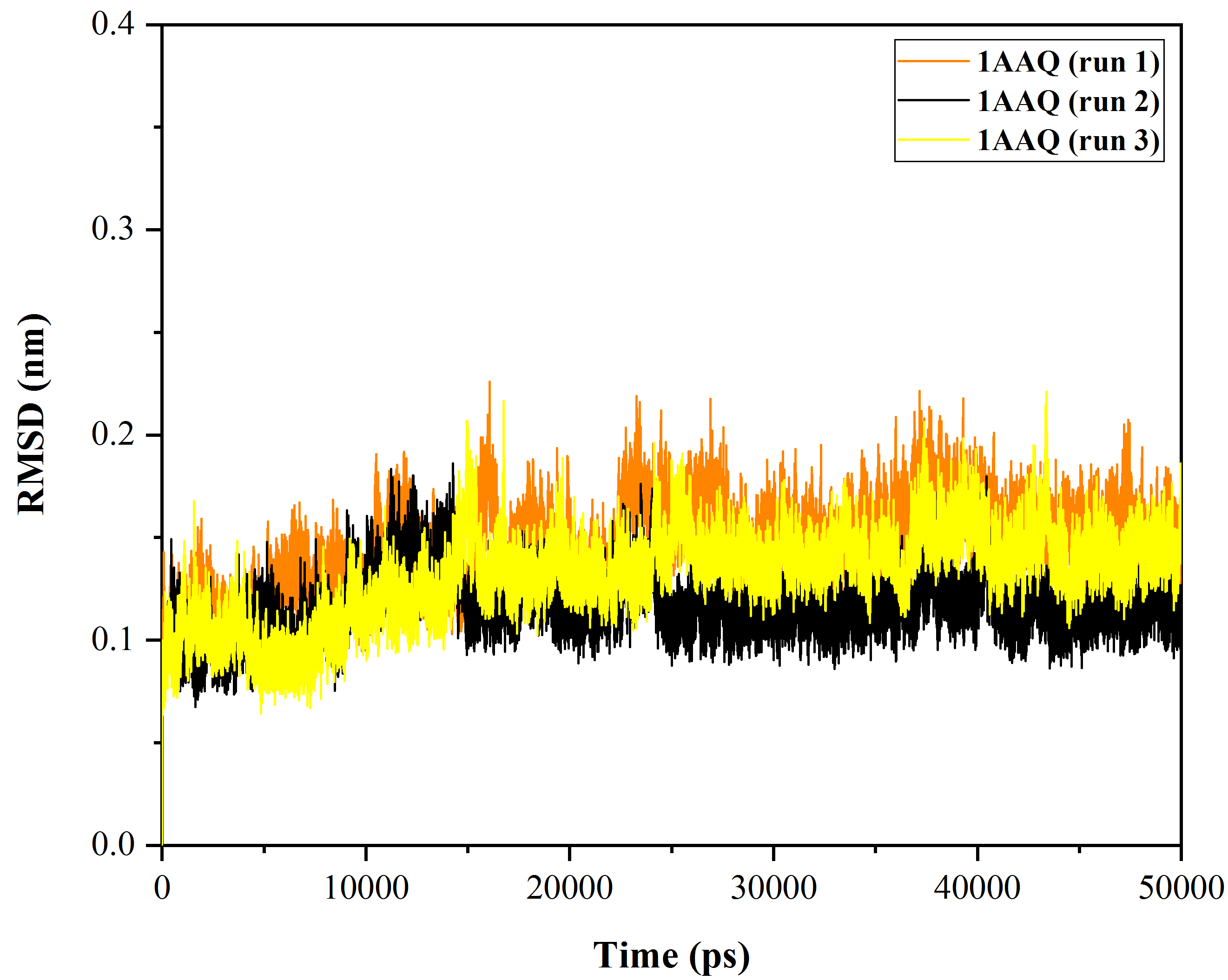


**Figure S3.** RMSD for HIV-1 protease protein backbone atoms (1AAQ code) for 3 repetitions of molecular dynamics simulations.


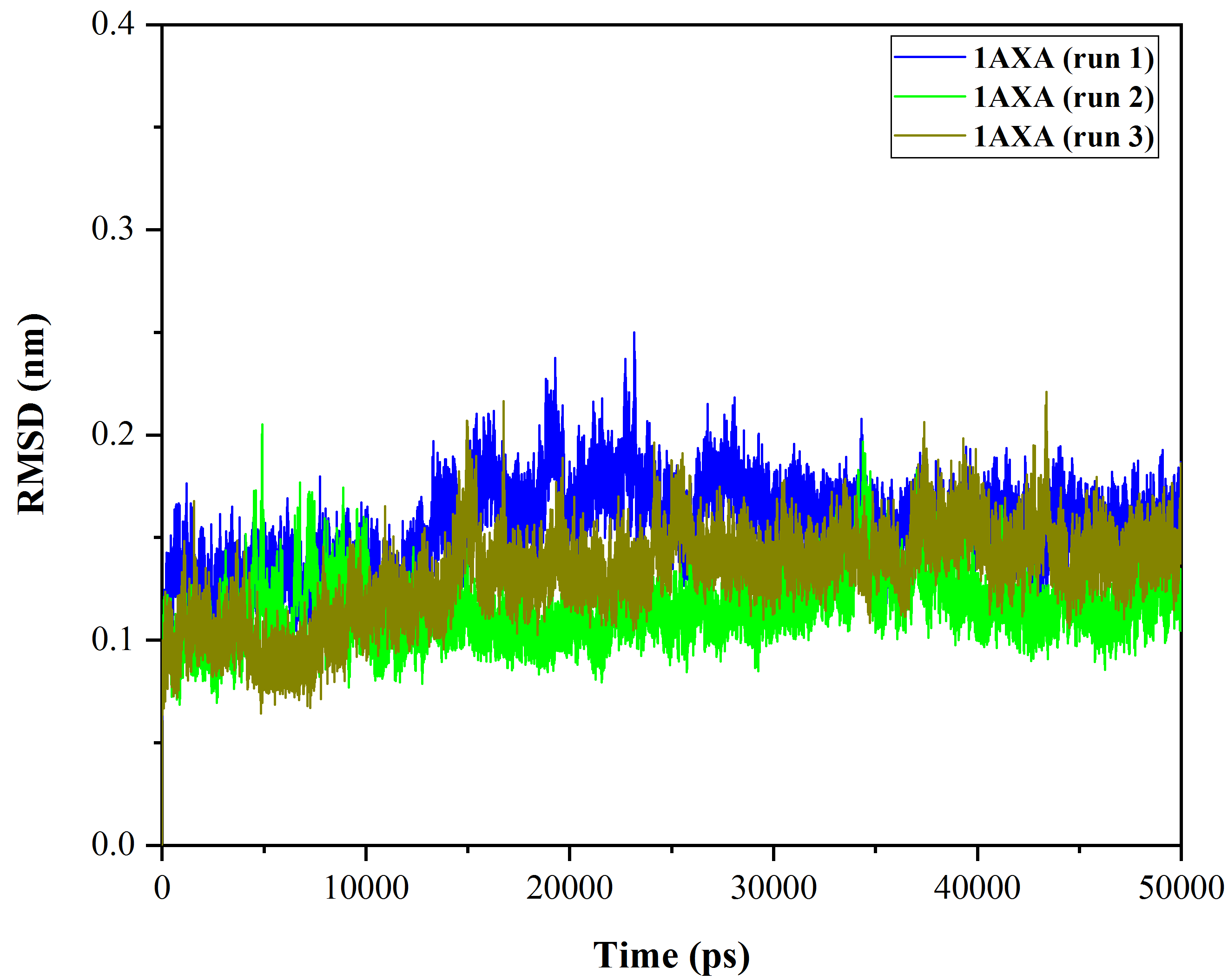


**Figure S4.** RMSD for HIV-1 protease protein backbone atoms (1AXA code) for 3 repetitions of molecular dynamics simulations.


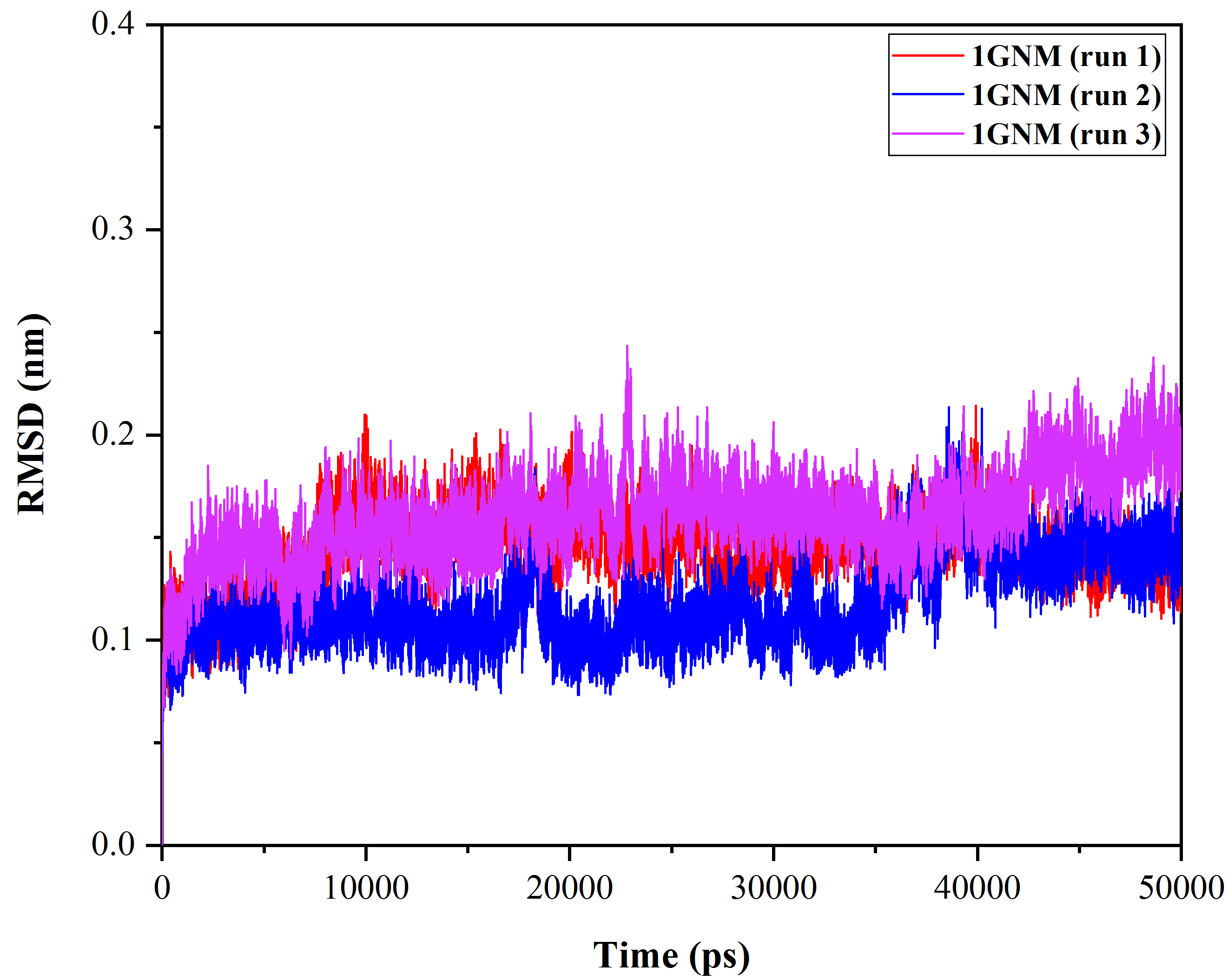


**Figure S5.** RMSD for HIV-1 protease protein backbone atoms (1GNM code) for 3 repetitions of molecular dynamics simulations.


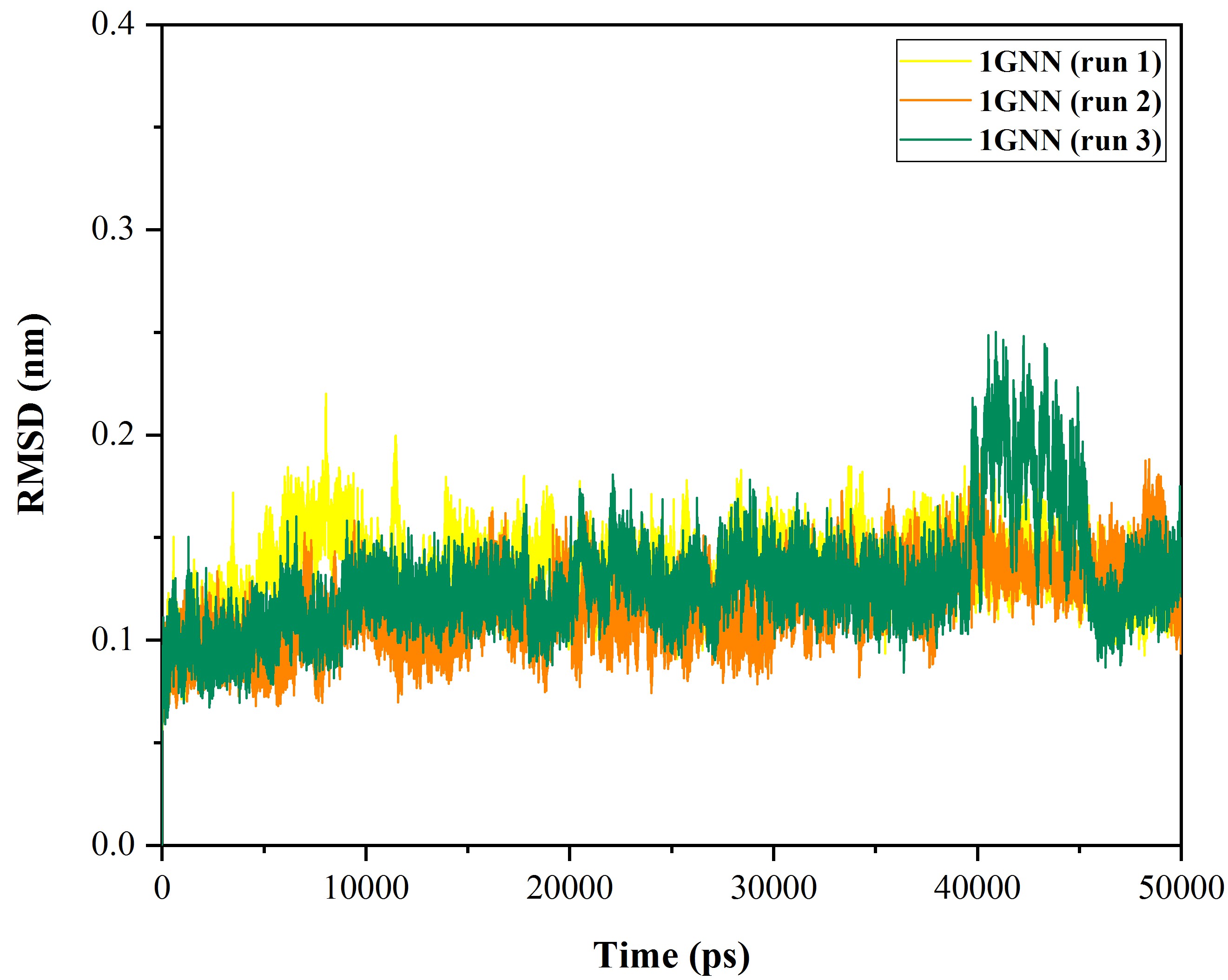


**Figure S6.** RMSD for HIV-1 protease protein backbone atoms (1GNN code) for 3 repetitions of molecular dynamics simulations.


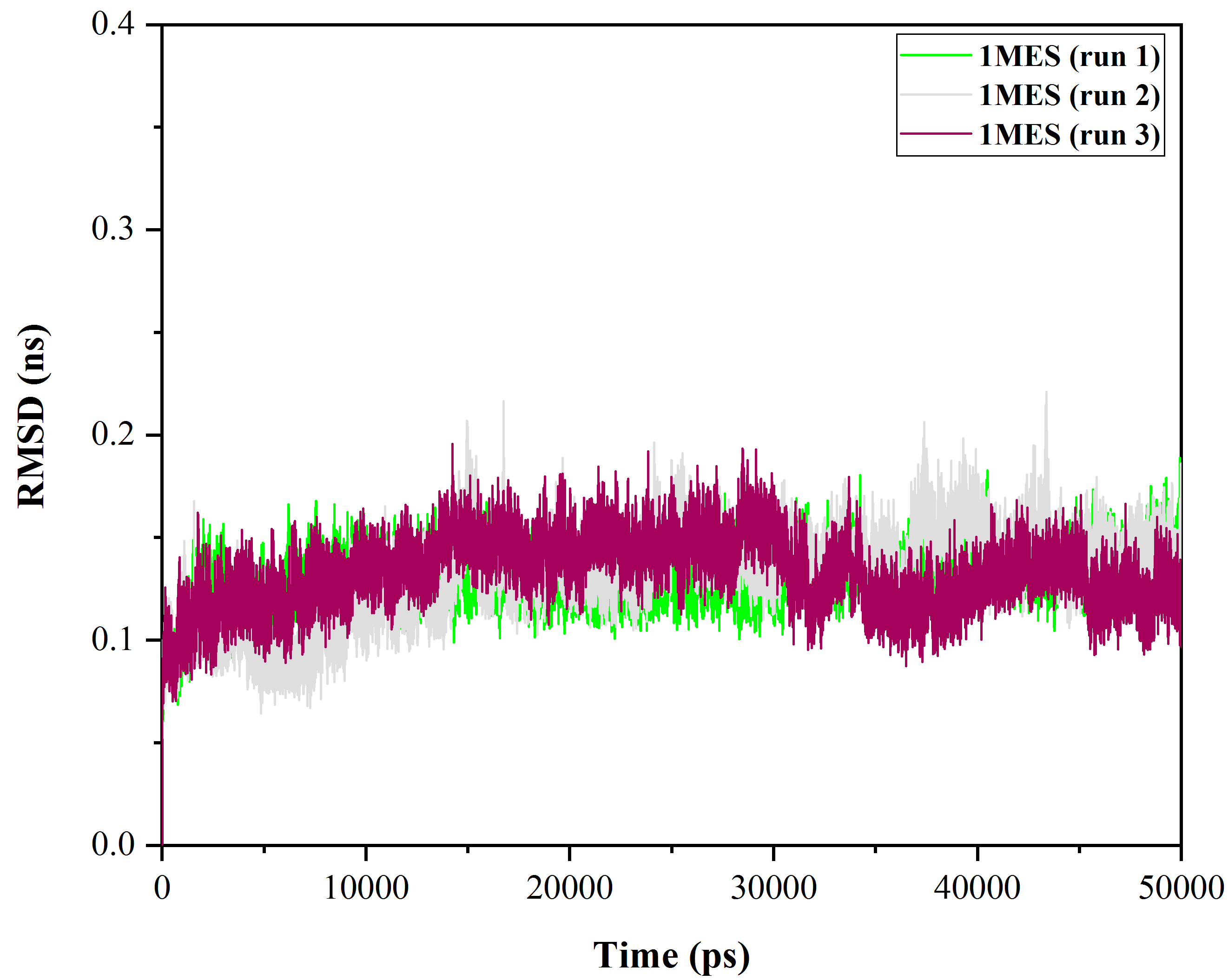


**Figure S7.** RMSD for HIV-1 protease protein backbone atoms (1MES code) for 3 repetitions of molecular dynamics simulations.


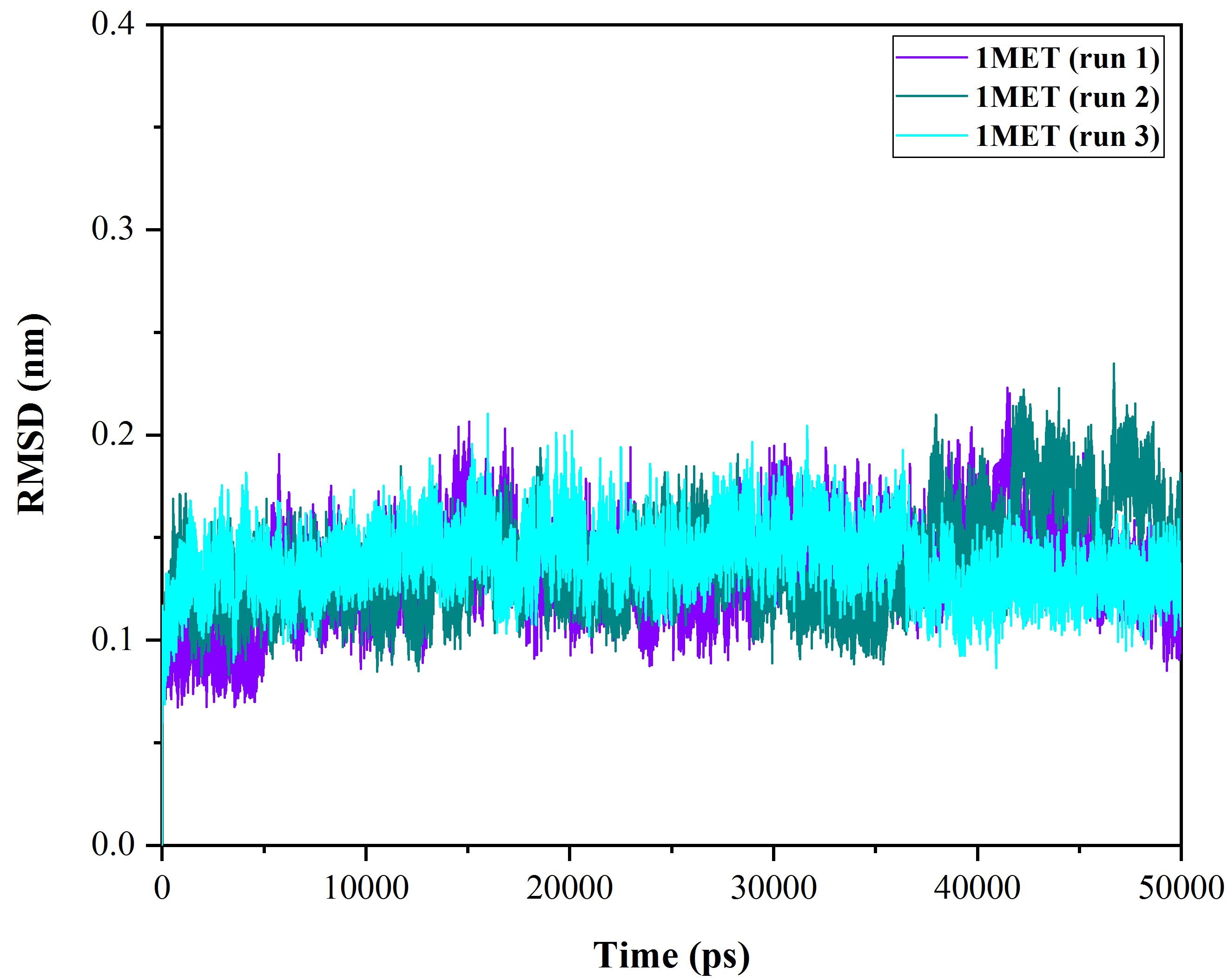


**Figure S8.** RMSD for HIV-1 protease protein backbone atoms (1MET code) for 3 repetitions of molecular dynamics simulations.


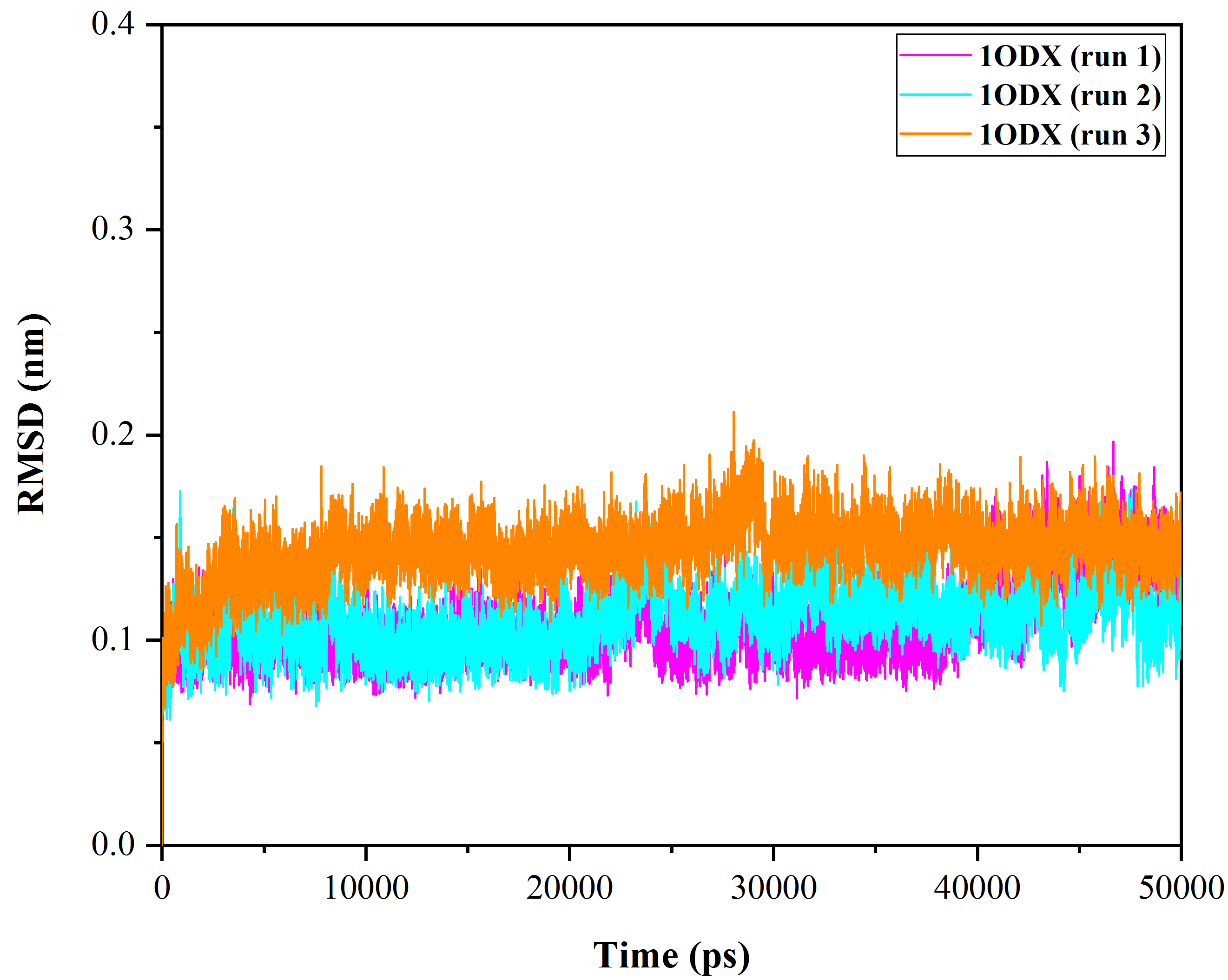


**Figure S9.** RMSD for HIV-1 protease protein backbone atoms (1ODX code) for 3 repetitions of molecular dynamics simulations.


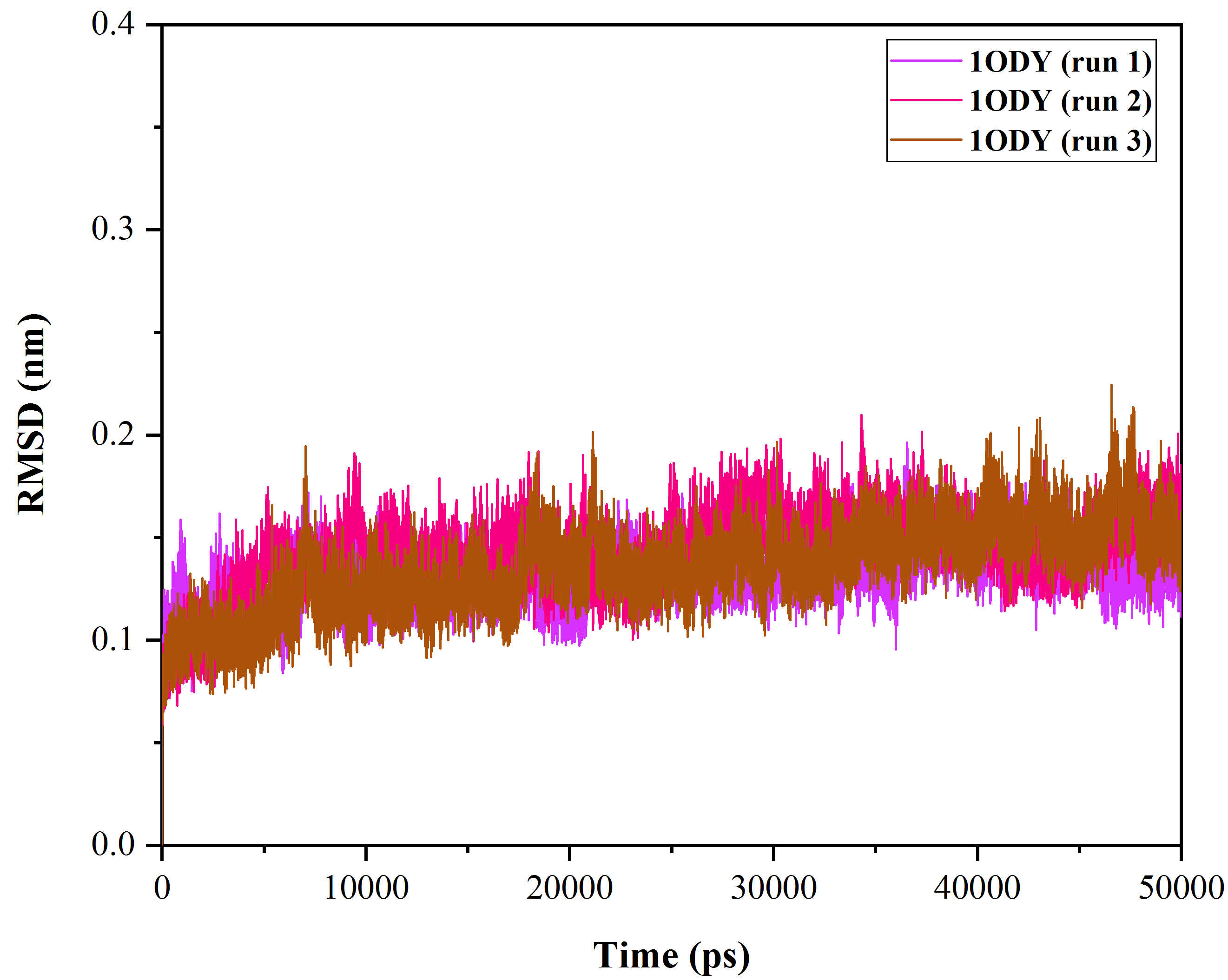


**Figure S10.** RMSD for HIV-1 protease protein backbone atoms (1ODY code) for 3 repetitions of molecular dynamics simulations.


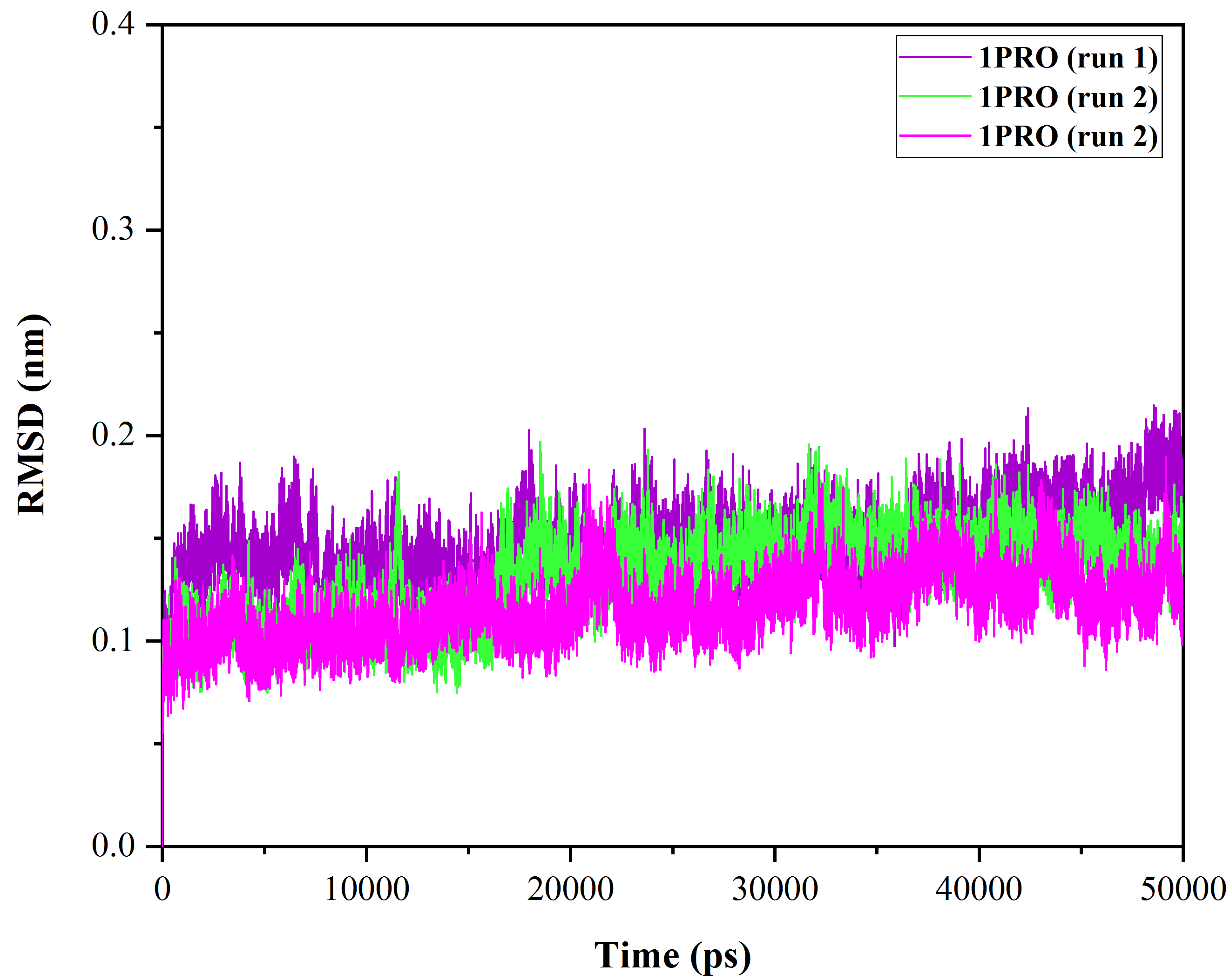


**Figure S11.** RMSD for HIV-1 protease protein backbone atoms (1PRO code) for 3 repetitions of molecular dynamics simulations.

**Table S1.** The cosine content of the principal components and the free energy values of the first two principal components were calculated by PCA and FEL analyses for each of the HIV-1 protease structures during 3 molecular dynamics simulations.

| **1HVI** | | | |
| --- | --- | --- | --- |
| **Run 3** | **Run 2** | **Run 1** |  |
| 0.005 | 0.582 | 0.542 | Cosine content PC1 |
| 0.002 | 0.074 | 0.003 | Cosine content PC1 and PC2 |
| 0 to 20.8 | 0 to 21.1 | 0 to 20.3 | Free energy (kJ/mol) |
| **1A9M** | | | |
| **Run 3** | **Run 2** | **Run 1** |  |
| 0.573 | 0.233 | 0.195 | Cosine content PC1 |
| 0.044 | 0.052 | 0.089 | Cosine content PC1 and PC2 |
| 0 to 22 | 0 to 19.7 | 0 to 20.6 | Free energy (kJ/mol) |
| **1AAQ** | | | |
| **Run 3** | **Run 2** | **Run 1** |  |
| 0.535 | 0.072 | 0.0634 | Cosine content PC1 |
| 0.039 | 0.145 | 0.138 | Cosine content PC1 and PC2 |
| 0 to 20.7 | 0 to 21 | 0 to 20.1 | Free energy (kJ/mol) |
| **1AXA** | | | |
| **Run 3** | **Run 2** | **Run 1** |  |
| 0.0224 | 0.239 | 0.840 | Cosine content PC1 |
| 0.379 | 0.265 | 0.006 | Cosine content PC1 and PC2 |
| 0 to 21 | 0 to 20.8 | 0 to 22.5 | Free energy (kJ/mol) |
| **1GNM** | | | |
| **Run 3** | **Run 2** | **Run 1** |  |
| 0.712 | 0.344 | 0.725 | Cosine content PC1 |
| 0.085 | 0.035 | 0.026 | Cosine content PC1 and PC2 |
| 0 to 21.4 | 0 to 20.1 | 0 to 19.7 | Free energy (kJ/mol) |
| **1GNN** | | | |
| **Run 3** | **Run 2** | **Run 1** |  |
| 0.419 | 0.738 | 0.064 | Cosine content PC1 |
| 0.142 | 0.0015 | 0.0003 | Cosine content PC1 and PC2 |
| 0 to 21.3 | 0 to 20.2 | 0 to 20.6 | Free energy (kJ/mol) |
| **1MES** | | | |
| **Run 3** | **Run 2** | **Run 1** |  |
| 0.199 | 0.406 | 0.612 | Cosine content PC1 |
| 0.007 | 0.004 | 0.0002 | Cosine content PC1 and PC2 |
| 0 to 19.7 | 0 to 20.1 | 0 to 20.3 | Free energy (kJ/mol) |
| **1MET** | | | |
| **Run 3** | **Run 2** | **Run 1** |  |
| 0.152 | 0.501 | 0.228 | Cosine content PC1 |
| 0.019 | 0.008 | 0.004 | Cosine content PC1 and PC2 |
| 0 to 19.7 | 0 to 20.3 | 0 to 20.1 | Free energy (kJ/mol) |
| **1ODX** | | | |
| **Run 3** | **Run 2** | **Run 1** |  |
| 0.0396 | 0.512 | 0.559 | Cosine content PC1 |
| 0.222 | 0.022 | 0.042 | Cosine content PC1 and PC2 |
| 0 to 20.3 | 0 to 20.1 | 0 to 20.2 | Free energy (kJ/mol) |
| **1ODY** | | | |
| **Run 3** | **Run 2** | **Run 1** |  |
| 0.254 | 0.614 | 0.599 | Cosine content PC1 |
| 0.271 | 0.0007 | 0.208 | Cosine content PC1 and PC2 |
| 0 to 20.8 | 0 to 20.1 | 0 to 19.6 | Free energy (kJ/mol) |
| **1PRO** | | | |
| **Run 3** | **Run 2** | **Run 1** |  |
| 0.830 | 0.140 | 0.518 | Cosine content PC1 |
| 0.0009 | 0.342 | 0.014 | Cosine content PC1 and PC2 |
| 0 to 20.6 | 0 to 20.5 | 0 to 20.1 | Free energy (kJ/mol) |
